# Gamified Adult Basic Life Support Training for Schoolchildren Using Minecraft Education Edition: Cross-Sectional Program Evaluation

**DOI:** 10.2196/93141

**Published:** 2026-09-09

**Authors:** Manuel Pardo Rios, Vanessa Fois González, Ana Belén Ocampo Cervantes, Carmen Amalia López López, José Tortosa Alcázar, Miriam Mendoza López, Petronila Mireia Alcázar Artero, Daniel Guillén Martínez, Robert Greif, Nino Fijačko, Aida Carballo-Fazanes, Cristian Abelairas-Gómez

**Affiliations:** 1 Grupo de Investigación de Nuevas Tecnologías para la Salud, Universidad Católica de Murcia, Av. de los Jeronimos, 135, Guadalupe, 30107, Spain, 34 968 27 88 08; 2 Gerencia de Urgencias y Emergencias Sanitarias 061 de la Región de Murcia, Murcia, Spain; 3 Faculty of Medicine, University of Bern, Bern, Switzerland; 4 Faculty of Health Sciences, University of Ljubljana, Ljubljana, Slovenia; 5 Faculty of Nursing, Universidade de Santiago de Compostela, Santiago de Compostela, Spain; 6 CLINURSID Research Group, Psychiatry, Radiology, Public Health, Nursing and Medicine Department, University of Santiago de Compostela, Santiago de Compostela, Spain; 7 Simulation and Intensive Care Unit of Santiago (SICRUS), Health Research Institute of Santiago, University Hospital of Santiago de Compostela - CHUS, Santiago Compostela, Spain; 8 Faculty of Education Sciences, Universidade de Santiago de Compostela, Santiago de Compostela, Spain

**Keywords:** cardiopulmonary resuscitation, basic life support, school-based training, gamification, serious games, Minecraft Education Edition

## Abstract

**Background:**

Early bystander cardiopulmonary resuscitation (CPR) and prompt defibrillation are key determinants of survival after out-of-hospital cardiac arrest, and school-based adult basic life support (BLS) education is a high-impact public health strategy. However, scalability and standardized assessment remain challenging. Gamified digital environments may sustain engagement while enabling comparable, rubric-based evaluation of learning artifacts. Few evaluations have examined whether a fully online gamified platform can support large-scale school-based adult BLS participation while generating standardized class-level artifacts for structured assessment.

**Objective:**

This study aims to assess the implementation of a fully online, school-oriented, gamified adult BLS initiative delivered through Minecraft Education Edition by quantifying program reach, rubric-based class-level project performance, and provider-side costs.

**Methods:**

We conducted a cross-sectional program evaluation of the 2025 Build & Save: CPR Museum Challenge during a 21-day window. Participation followed open, voluntary, convenience-based, self-selected sampling in schools. Teachers facilitated a self-directed online adult BLS course and a structured in-game building task with rooms representing key adult BLS actions (checking responsiveness, calling 112, compressing, defibrillating). Each class submitted one project (diary and world file), the unit of analysis; teacher and student counts were recorded only as reach indicators. Eligible submissions included the required PDF diary and an accessible world file; incomplete or nonassessable submissions were excluded before scoring. A 4-member jury assessed projects using a predefined 5-domain rubric (score of 1‐4 per domain; base score 0‐20; total 0‐21, capped at 20). Descriptive statistics, Mann-Whitney *U* tests, Kruskal-Wallis tests, Spearman correlations, item-total reliability, and Cronbach α were computed using a 2-sided α of .05. Inferential tests were interpreted as exploratory. A cost-consequence analysis reported provider-side costs.

**Results:**

Thirty class-level projects were submitted; 27 were evaluated after excluding incomplete or nonassessable submissions. The initiative reached 23 teachers and 850 students across 11 regions (Spain, United States, Colombia, Malaysia). The median base and total scores were 7 (IQR 2‐10; range 0‐20). Rubric internal consistency was high (Cronbach α=0.95, 95% CI 0.89-0.97), with corrected item-total correlations of 0.81‐0.89. Projects including an explanatory video scored higher than those without a video (Mann-Whitney *U*=134.5; *P*=.006; Cliff delta=0.66, 95% CI 0.37‐0.92), and secondary-level projects outperformed primary-level submissions (Kruskal-Wallis *H*=7.46; *P*=.006). Domain correlations were strong (all *P*<.001; highest ρ=0.94). Direct implementation costs were €4500 (US $5126), rising to €6000 (US $6835) including awards; the cost per student ranged from €5.29 (US $6.03) to €7.06 (US $8.04).

**Conclusions:**

This single-edition program evaluation suggests that a teacher-facilitated, low-cost, Minecraft-based school initiative may support wide reach and scalable, standardized assessment through class-level artifacts, but it does not demonstrate educational effectiveness in CPR knowledge or skills. Stronger instructional scaffolding is needed, particularly in primary education. Future controlled studies should evaluate knowledge and skill acquisition, retention, and transfer to real-world bystander behavior. Digital gamified activities should complement, not replace, hands-on CPR training.

## Introduction

### Background

Early recognition, immediate bystander cardiopulmonary resuscitation (CPR), and prompt defibrillation are critical determinants of survival in out-of-hospital cardiac arrest [1]. Although the Formula of Survival is well established in international consensus [1-3], the challenge remains in the implementation factor, which means translating scientific evidence into effective community action. Recent evidence suggests that single, traditional instructor-led CPR courses, even when initially effective, are not enough to overcome the attrition of CPR skills: in laypeople, compression and ventilation performance often deteriorate within 3 to 6 months after training [4,5]. These findings, together with data showing that 6-month or more frequent refresher sessions (including blended and/or low-dose or high-frequency formats) better sustain high-quality CPR than longer intervals, support calls for periodic, skill-focused retraining rather than relying on isolated, on-time, instructor-led courses [6-10]. Consequently, current international recommendations emphasize the need for contemporary educational designs and technology-enhanced learning, especially when traditional instructor-led training is difficult to scale at a population level [11,12].

This need for scalability aligns directly with the “Kids Save Lives” framework, pushing for school-based adult basic life support (BLS) education as a high-impact public health strategy [12]. Although schoolteachers are considered key instructors, they usually do not feel confident enough to teach due to a lack of knowledge and training [13]. Other barriers to the successful integration of BLS into school curricula are time constraints and varying levels of student engagement. To address these hurdles, digital tools, including social media, mobile apps, and gamified platforms, have emerged as essential assets for supporting and assessing learning at scale [12]. Gamification, defined as the use of game-design elements in nongame contexts, has moved from a novel trend to a core pedagogical strategy, with recent research highlighting its ability to enhance intrinsic motivation and long-term knowledge retention in medical education [14,15]. Specifically, the use of serious games allows for challenge-based learning in which students can practice high-stakes decision-making in a risk-free environment [16,17]. Among these platforms, Minecraft Education Edition provides a unique sandbox environment that is familiar and immersive, fostering collaboration and authentic problem-solving.

The mechanisms through which gamified environments may foster learning are increasingly described. From a self-determination perspective, game-design elements can support the basic psychological needs for autonomy, competence, and relatedness that underpin intrinsic motivation [18]. Work on gamification affordances further suggests that progress, psychological empowerment, hedonic well-being, and inspiration are proximal drivers of engagement and behavior change in digital health applications [19]. In a building-based challenge such as the present one, these affordances are expected to elicit high engagement and creative output; however, when instructional scaffolding is weak, the same ludic affordances may draw attention toward esthetic and narrative elements rather than the accurate representation of clinical content, a tension we examine through domain-specific rubric scores. Building on prior school-based serious-game work, which has mostly evaluated individual skill acquisition in single settings [20,21], the present study addresses a complementary gap: whether a fully online format can couple large-scale participation with standardized, rubric-assessable class-level artifacts.

Beyond engagement, such platforms can function as standardized evaluation tools, generating comparable learning artifacts (student-built projects), standardized rubric-based performance indicators, and participation metrics at scale. Accordingly, this study addressed the following research questions: (1) What program reach could be achieved through a fully online, school-based Minecraft Education Edition adult BLS challenge? (2) What level and pattern of rubric-based performance were observed in the submitted class-level projects? (3) What were the direct provider-side costs and unit costs of implementing the 2025 edition of the initiative?

### Objective

The objective of this cross-sectional program evaluation was to assess the implementation of a gamified, school-oriented adult BLS training initiative delivered through Minecraft Education Edition by quantifying program reach, rubric-based class-level project performance, and provider-side costs to inform future implementation and scale-up [1-3,11,12].

## Methods

### Study Design and Setting

We conducted a cross-sectional evaluation of a school-based adult BLS educational program. The initiative was delivered fully online during the 2025 edition of the Build & Save: CPR Museum Challenge. Participation took place within a 21-day window (September 22 to October 13, 2025), culminating with an online award event on World Restart a Heart Day (October 16, 2025). Quantitative outcomes included reach or engagement indicators and rubric-based project scores (see below). Additionally, a cost-consequence analysis was performed from the program-provider perspective using direct program costs from the 2025 edition [22].

### Educational Intervention and Challenge Format

#### Educational Intervention

The educational initiative was designed as a school-based, fully online CPR program linked to World Restart a Heart Day (October 16, 2025). All educational resources, registration instructions, and downloadable materials were hosted on the official Challenge webpage [23] (Multimedia Appendix 1 and Multimedia Appendix 2).

The intervention combined (1) self-directed adult BLS learning through Fundación MAPFRE’s online course “Aprendiendo a salvar vidas con Minecraft” (link to website program [23]) and (2) a structured Minecraft Education Edition building challenge (“Build & Save: CPR Museum”), in which students created an in-game museum with dedicated rooms representing adult BLS actions [3]: check responsiveness, call for help (emergency number 112), compress, and defibrillate, following standardized teacher guides and a predefined rubric. The teacher guides provided step-by-step instructions and examples for each room (eg, use of signs or boards and nonplayer characters to convey messages and to link to external educational resources, and optional the use of redstone or automation elements).

#### Challenge Format and Implementation

Teachers could organize participation as a whole class or in small groups (recommended up to 5 students per group), considering technical limitations (Minecraft Education allows up to 40 simultaneous users in the same world). Only 1 project per participating class could be submitted by the teacher, which included (1) a project diary PDF with screenshots and explanations of the proposal and (2) the exported Minecraft world (.mcworld), stored in a cloud folder with public view permissions and shared via download link. An optional explanatory video (≤2 min) could be submitted for an additional point in the final score.

#### Timeline and Incentives

Registrations were open from September 22, 2025, to October 13, 2025. The initiative ended in a final online event on October 16, 2025, coinciding with World Restart a Heart Day, with the announcement of the winners; the top 3 projects each received a €500 (€1=US $1.15 as of August 3, 2026) school-supplies award in school supplies. All participating teachers and students received certificates of participation.

### Recruitment and Sampling

Participation was based on open voluntary registration in the 2025 edition of the Build & Save: CPR Museum Challenge. All educational resources, registration instructions, and downloadable materials were made available through the official Challenge webpage. The target setting was school-based participation, and teachers acted as facilitators of the activity within their classrooms. The sampling approach was nonprobability convenience sampling based on open voluntary self-registration by teachers and schools during the recruitment window (September 22 to October 13, 2025). The unit of participation was the school class, and each teacher could submit a maximum of 1 project per participating class.

### Eligibility and Exclusion Criteria

Eligible submissions were class-level projects submitted by a teacher during the 2025 challenge period and consisting of the required deliverables: (1) a project diary in PDF format and (2) an exported Minecraft world file (.mcworld) accessible through a download link. An optional explanatory video (≤2 min) could also be submitted for a bonus point in the final score, but it was not required for eligibility. Submissions were excluded from formal evaluation if they lacked one or more required deliverables, could not be accessed or opened for technical reasons, or contained insufficient material to allow rubric-based assessment of the predefined domains. These criteria were applied before scoring.

### Data Sources and Outcome Measures

Data were obtained from the project submissions and the program implementation records. Each class-level submission included a project diary (PDF) with screenshots and explanatory text and an exported Minecraft world file (.mcworld); an optional explanatory video could also be provided. The primary quantitative outcomes were rubric-based project scores across 5 predefined domains: creativity and originality, technology and mechanisms, consistency with the CPR theme, integration of educational content in the museum, and clarity of in-world explanations. Additional descriptive outcomes included participation and reach indicators (number of submitted and evaluated projects, teachers, students, educational level, and geographic origin). A secondary outcome was the total project score including the optional video bonus point. Economic outcomes included direct implementation costs, awards, and unit costs per teacher, student, and project from the program-provider perspective.

### Rubric Scoring Procedure

All 27 eligible class-level projects were reviewed by the full 4-member Challenge jury using the predefined rubric (Table 1), which comprised 5 domains: creativity and originality, technology and mechanisms, consistency with the CPR theme, integration of CPR educational content in the museum, and clarity of in-world explanations. Each domain was scored from 1 to 4, and the base score was calculated as the sum of the 5 domains; an additional bonus point was added when an optional explanatory video was submitted.

The jury was composed of 2 Fundación MAPFRE adult BLS instructors and 2 L3Tcraft Educación specialists in Minecraft Education Edition. Before formal scoring, the jury held a calibration session to review the rubric criteria and harmonize the interpretation of each domain (Table 2). Each juror first completed an individual assessment of every project, after which the final domain scores were assigned through consensus discussion among the 4 jury members. Because the final scores were determined by consensus and the preliminary individual ratings were not retained, formal interrater reliability was not calculated.

**Table 1.** Rubric used in this cross-sectional program evaluation of the 2025 Build & Save: CPR Museum Challenge, a fully online school-based adult BLS^a^ educational initiative delivered through Minecraft Education Edition.^b^

Domains	Rating			
	1	2	3	4
Creativity and originality	The construction is basic and follows a common design.	The construction shows some creativity but remains simple.	The building is creative and has original elements.	The building is very creative and unique, with many original elements.
Technology and mechanisms (redstone, command blocks…)	No advanced technologies are used.	Some basic technologies and mechanisms are used.	Several advanced technologies and mechanisms are used.	Many advanced technologies and mechanisms are used, including innovative automation systems.
Consistency with the theme (CPR^c^ rooms, adult BLS steps)	Weak or nonexistent reflection of CPR steps.	Reflection present but not entirely clear with the CPR steps.	Reflection present and clearly shown for some CPR steps.	Each room clearly reflects a CPR step and its importance (symbols, NPCs^d^, explained processes, etc).
Integration of content in the CPR museum (eg, boards or NPCs)	The world contains no content elements such as boards or NPCs.	The world contains some content such as boards or NPCs.	The world contains several types of content such as boards or NPCs.	The world contains many types of content such as boards or NPCs.
Explanation of content in the world	The world explanation is poor and confusing.	The explanation within the world is acceptable and clear in some aspects.	The explanation of the world’s elements is good and clear, with some coherence between items such as boards and NPCs.	The explanation of the world’s elements is excellent, very clear and detailed, with high coherence between items such as boards and NPCs.

a BLS: basic life support.

b The rubric was applied to class-level submissions from participating schoolchildren and assessed 5 domains: creativity and originality, technology and mechanisms, consistency with the cardiopulmonary resuscitation theme, integration of educational content, and clarity of in-world explanations.

c CPR: cardiopulmonary resuscitation.

d NPC: nonplayer character.

**Table 2.** Study variables and measures used in this cross-sectional program evaluation of the 2025 Build & Save: CPR Museum Challenge (Minecraft Education Edition).^a^

Variable	Type	Operational definition	Scale or range	Data source
Participating teachers	Reach (count)	Teachers submitting at least one class project	Count	Program records
Participating students	Reach (count)	Students reached (teacher-reported)	Count	Program records
Regions or countries	Reach (categorical)	Geographic origin of submissions	11 regions	Program records
Educational level	Independent (categorical)	Primary vs secondary education of the class	2 levels	Project submission
Explanatory video	Independent (binary)	Optional video (≤2 min) submitted	Yes or no (+1 bonus)	Project submission
Creativity and originality	Outcome (ordinal)	Rubric domain 1	1‐4	Jury rubric (Table 1)
Technology and mechanisms	Outcome (ordinal)	Rubric domain 2	1‐4	Jury rubric (Table 1)
Consistency with CPR^b^ theme	Outcome (ordinal)	Rubric domain 3	1‐4	Jury rubric (Table 1)
Content integration	Outcome (ordinal)	Rubric domain 4	1‐4	Jury rubric (Table 1)
In-world explanation clarity	Outcome (ordinal)	Rubric domain 5	1‐4	Jury rubric (Table 1)
Base score	Primary outcome	Sum of the 5 rubric domains	0‐20	Derived
Total score	Secondary outcome	Base score plus video bonus	0‐21 (capped 20)	Derived
Implementation cost	Economic	Direct provider-side delivery cost	Euro	Program accounts
Total expenditure	Economic	Implementation cost plus awards	Euro	Program accounts
Unit costs	Economic	Cost per teacher or student or project	Euro	Derived

a The measurement items of the scored construct (the 5 rubric domains and their 1‐4 anchors) are detailed in Table 1.

b CPR: cardiopulmonary resuscitation.

### Economic Evaluation

A cost-consequence analysis was conducted from the program-provider (organizer) perspective, reporting monetary values in euros excluding value-added tax. Direct implementation costs for delivering the 2025 edition (including world adaptation, website or communication materials, contest design and rubric-based evaluation, and participant support) were €4500. Provided awards added another €1500 (3 prizes of €500 in school supplies). Total expenditure was €6000.

For scalability or sensitivity reporting, unit costs were calculated under 2 scenarios, implementation costs €4500, and total expenditure €6000, divided each by key program outputs (eg, cost per participating teacher, per participating student, and per submitted or evaluated project) using denominators from the final report. Teacher classroom time was not monetized, as participation was integrated into routine school hours and delivered by teachers as part of their usual teaching activities.

### Ethical Considerations

The study was conducted in accordance with the ethical principles of the Declaration of Helsinki and was approved by the UCAM (Universidad Católica de Murcia) Research Ethics Committee (CE112309). Institutional consent at the school or classroom level for participation in the initiative and for the use of the generated project data for research purposes was obtained electronically at the start of the online experience. Because submissions were made by teachers on behalf of the participating classes and no individually identifiable data from minors were collected, the ethics committee approved the use of these anonymized class-level project data for research purposes without requiring additional individual written consent from students. No financial compensation was provided to participants; the initiative offered participation certificates to all participating teachers and students, and school-supply awards were granted to the top 3 projects as part of the challenge format rather than as compensation for research participation. No identifiable images of individual participants were included in the manuscript or supplementary materials.

### Statistical Analyses

All quantitative analyses were conducted at the project level, using class-level submissions as the unit of analysis. All statistical analyses were performed using Python (v3.10; Python Software Foundation) with relevant scientific libraries. Descriptive statistics were used to summarize participation metrics and rubric-based project scores and are presented as medians and IQRs due to nonnormal score distributions. Teacher and student counts were summarized descriptively as indicators of program reach and were not treated as independent analytical observations. A 2-sided α level of .05 was used for inferential analyses. To assess differences in project quality, nonparametric tests were applied. The Mann-Whitney *U* test was used to compare scores between projects with and without explanatory videos. Effect size was estimated using the Cliff delta, with 95% CIs computed via bootstrapping. Positive δ indicates higher values in the first group, and negative δ indicates higher values in the second group. The Kruskal-Wallis test was used to compare base scores across educational levels (primary vs secondary school), followed by post hoc pairwise comparisons using the Mann-Whitney *U* test with Holm correction when appropriate. Spearman rank correlation coefficients were calculated to explore associations between rubric domains, and 95% CIs were reported for the main correlation estimates. Internal consistency across the 5 rubric items was assessed using Cronbach α, with 95% CIs. For relevant proportions, absolute values (n/N), percentages, and 95% CIs were reported. Unit costs per participant group were calculated by dividing total program costs by the number of participating teachers, students, and projects under 2 cost scenarios (implementation only vs full program with awards); these costs were reported descriptively as observed program values.

## Results

### Overview

A total of 30 class-level projects were submitted to the 2025 CPR Challenge from 11 regions (7 regions in Spain, 2 US states, Colombia, and Malaysia). Of these, 3 submissions were excluded, leaving 27 class-level projects for formal evaluation. These participating classes represented 23 teachers and approximately 850 students; however, all quantitative analyses were performed on the 27 evaluated project submissions, with teacher and student counts reported only as indicators of program reach. A recording of the event is publicly available online [24].

### Project Scores

Table 3 summarizes the project-level dataset. Of the 27 evaluated projects, 14 (51.9%) were from primary education and 13 (48.1%) from secondary education. Nine (33.3%) projects included the optional explanatory video, qualifying for a+1 bonus point in the final score. Geographically, the final sample was predominately Spanish, with the exception of 2 projects from Malaysia.

**Table 3.** Project-level rubric scores for the 27 evaluated class submissions included in this cross-sectional program evaluation of the 2025 Build & Save: CPR Museum Challenge, a fully online school-based adult basic life support (BLS) initiative delivered through Minecraft Education Edition during September to October 2025.

Center name	Project name	Course	Video (yes or no)	Domains, score					Total score
				Creativity and originality	Technology and mechanisms	Coherence with the theme	Integration of contents	Explanation of contents	
1. FEC Providencia Sagrado Corazón	Museo de la RCP.	4° ESO^a^	Yes	4	4	4	4	3	20
2. Colegio Jesus Maria	GRUPO LOS MALEANTES	4° ESO	No	4	4	3	3	2	16
3. Cantera de Empresas Xativa	Museo RCP Cantera de Empresas Xàtiva	5° Primaria	Yes	3	2	3	2	3	14
4. GSD Las Rozas	Museo RCP Minecraft Academy GSD Las Rozas	4° Primaria	Yes	2	2	2	2	3	12
5. Colegio Jesus Maria	LOS INHUMANOS	4° ESO	No	3	2	3	2	2	12
6. Colegios Educare Antanes School	Hospital IHET	6° Primaria	Yes	3	2	3	2	1	12
7. Sekolah Datuk Akhir Zaman	Malaysian Winners CPR Museum	Grade 8, 9, 10, and 11	Yes	2	1	2	2	2	10
8. Sekolah Datuk Akhir Zaman	CPR MUSEUM	Grade 8, 9, 10, and 11	Yes	2	1	2	2	2	10
9. Colegio Jesus Maria	NOSE	4° ESO	No	2	2	2	2	2	10
10. Colegio Nuestra Señora Del Huerto	MUNDO RCP COLEGIO NUESTRA SEÑORA DEL HUERTO 2° ESO	2° ESO	Yes	2	2	2	1	1	9
11. Colegio Jesus Maria	SIRI	4° ESO	No	2	1	3	1	1	8
12. Maristak Zalla	RCP “Rápido, claro y positivo” Rapidamente hay que actuar con claridad para un resultado positivo	2° ESO	Yes	1	2	2	1	1	8
13. Ntra. Sra. de la Salud - Maristas Algemesí	Hospital Sanguíneo	3° ESO	No	2	1	2	1	1	7
14. Cantera de Empresas Xativa	Museo RCP Xàtiva L	3° Primaria	No	2	1	2	1	1	7
15. Ntra. Sra. de la Salud - Maristas Algemesí	Hospital Marista	3° ESO	Yes	1	0	0	2	1	5
16. IES Antoni Maura	Mi mundo RCP	3° ESO	No	1	1	1	1	1	5
17. IES Antoni Maura	STEAM world challenge	3° ESO	No	1	1	1	1	1	5
18. CEIP^b^ Jacinto Benvaente	JBA 299	5° Primaria	No	1	0	0	1	1	3
19. CEIP Jacinto Benvaente	JBA 374	6° Primaria	No	1	0	0	1	1	3
20. CEIP Jacinto Benvaente	JBA 412	5° Primaria	No	1	2	0	0	0	3
21. CEIP Jacinto Benvaente	JBA 407	6° Primaria	No	2	0	0	0	0	2
22. CEIP Jacinto Benvaente	JBA 308	5° Primaria	No	1	0	0	0	0	1
23. CEIP Jacinto Benvaente	JBA 396	6° Primaria	No	0	1	0	0	0	1
24. CEIP Jacinto Benvaente	JBA 415	5° Primaria	No	1	0	0	0	0	1
25. CEIP Jacinto Benvaente	JBA 438	6° Primaria	No	1	0	0	0	0	1
26. CEIP José Saramago	Salvando vidas	5° Primaria	No	1	0	0	0	0	1
27. CEIP José Saramago	RCP Primeros auxilios	5° Primaria	No	0	0	0	0	0	0

a ESO: Educación Secundaria Obligatoria (Compulsory Secondary Education).

b CEIP: Colegio de Educación Infantil y Primaria (Infant and Primary Education School).

Rubric-scored evaluation results are summarized in Table 4. Recalculation of the 27 evaluated class-level submissions showed a median base score (sum of the 5 rubric domains) of 7 (IQR 2‐10; range 0‐19). Total scores, which included a 1-point bonus for projects with an explanatory video, ranged from 0 to 20, with a median of 7 (IQR 2‐10). The proportion of projects receiving high scores (defined as 3 or 4 points) varied across domains, from 2/27 (7.4%, 95% CI 0.9%‐24.3%) in the Technology and mechanisms and Content integration domains to 6/27 (22.2%, 95% CI 8.6%‐42.3%) in the Consistency with CPR theme domain. Internal consistency across the 5 rubric domains was high (Cronbach *α*=0.95, 95% CI 0.89-0.97).

**Table 4.** Domain-level rubric score distribution across the 27 evaluated class submissions in this cross-sectional program evaluation of a fully online school-based adult basic life support initiative delivered through Minecraft Education Edition (2025 Build & Save: CPR Museum Challenge, September-October 2025).

Domains	Median (IQR)	High scores, n (%; 95% CI)
Creativity and originality	2 (1‐2)	5 (18.5%; 6.3%‐38.1%)
Technology and mechanisms	1 (0‐2)	2 (7.4%; 0.9%‐24.3%)
Consistency with CPR^a^ theme	2 (0‐2)	6 (22.2%; 8.6%‐42.3%)
Content integration	1 (0‐2)	2 (7.4%; 0.9%‐24.3%)
In-world explanation clarity	1 (0‐2)	3 (11.1%; 2.4%‐29.2%)

a CPR: cardiopulmonary resuscitation.

In a reliability analysis of the 5 rubric domains, corrected item-total correlations ranged from 0.81 to 0.89, and Cronbach α if an item was deleted ranged from 0.93 to 0.94, never exceeding the overall α; no domain improved reliability upon deletion; the corresponding interdomain correlations are shown in Table 5. In exploratory univariable linear regressions of the base score, video submission (*b*=5.33; *R*^2^=0.26) and secondary educational level (*b*=5.01; *R*^2^=0.26) were each associated with higher scores, consistent with the nonparametric tests.

**Figure 1.**
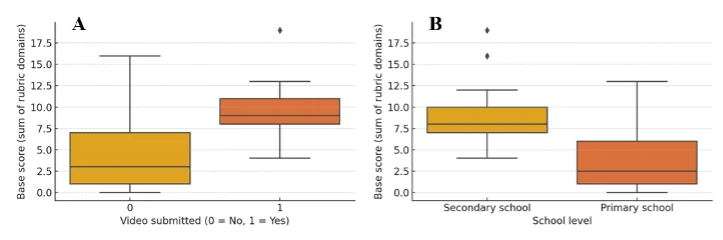
Box plots of base scores (sum of the 5 rubric domains) across submission characteristics in this cross-sectional program evaluation of the 2025 Build & Save: CPR Museum Challenge, a fully online school-based adult basic life support (BLS) initiative delivered through Minecraft Education Edition. Panel A compares projects with and without an explanatory video. Panel B compares primary- and secondary-level submissions. The center line indicates the median, the box indicates the IQR, and the whiskers indicate the full observed range.

Projects that included an explanatory video had higher base scores than those without a video (Mann-Whitney *U*=134.5, *P*=.006), with a moderate-to-large effect size (Cliff *δ*=0.66, 95% CI 0.37‐0.92). This is illustrated in Figure 1A. Base scores also differed by educational level (Kruskal-Wallis *H*=7.46; *P*=.006), and post hoc testing showed that secondary-level submissions scored significantly higher than primary-level submissions (Holm-adjusted *P*=.007). This is shown in Figure 1B.

**Figure 2.**
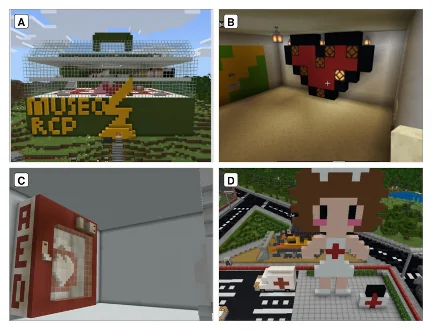
Representative in-game screenshots from class-level projects submitted to the 2025 Build & Save: CPR Museum Challenge, a fully online school-based adult basic life support (BLS) initiative delivered through Minecraft Education Edition (September-October 2025). (A) Museum entrance and identity; (B) themed room depicting the BLS chain; (C) automated external defibrillator (AED) room representing the defibrillation step; (D) nonplayer character (NPC) used to convey educational content within the world. No identifiable images of participants are shown.

Representative in-game screenshots of submitted CPR-museum projects are provided in Figure 2.

Spearman correlations between rubric domains were consistently positive and statistically significant (all *P*<.001; Table 5). Correlation coefficients ranged from ρ of 0.65 (95% CI 0.32‐0.88) for Technology and mechanisms versus Content integration to ρ of 0.94 (95% CI 0.85‐0.99) for Content integration versus In-world explanation clarity, indicating substantial overlap among several rubric dimensions.

**Table 5.** Pairwise Spearman correlations between rubric domains across the 27 evaluated class submissions in this cross-sectional program evaluation of the 2025 Build & Save: CPR Museum Challenge, a fully online school-based adult basic life support initiative delivered through Minecraft Education Edition during September to October 2025.

Domain 1	Domain 2	Spearman ρ (95% CI)	*P* value
Creativity and originality	Technology and mechanisms	0.67 (0.38-0.88)	<.001
Creativity and originality	Consistency with CPR^a^ theme	0.87 (0.69-0.96)	<.001
Creativity and originality	Content integration	0.75 (0.51-0.90)	<.001
Creativity and originality	In-world explanation clarity	0.73 (0.52-0.86)	<.001
Technology and mechanisms	Consistency with CPR theme	0.81 (0.57-0.95)	<.001
Technology and mechanisms	Content integration	0.65 (0.32-0.88)	<.001
Technology and mechanisms	In-world explanation clarity	0.67 (0.38-0.86)	<.001
Consistency with CPR theme	Content integration	0.78 (0.53-0.93)	<.001
Consistency with CPR theme	In-world explanation clarity	0.79 (0.61-0.91)	<.001
Content integration	In-world explanation clarity	0.94 (0.85-0.99)	<.001

a CPR: cardiopulmonary resuscitation.

### Cost and Cost Per Unit

Table 6 illustrates a relatively low cost through this digital format per unit under 2 scenarios: scenario A: direct implementation costs only (€4500) and scenario B: total costs including awards (€6000).

**Table 6.** Provider-side unit cost estimates for the 2025 Build & Save: CPR Museum Challenge reported in this cross-sectional program evaluation of a fully online school-based adult basic life support initiative delivered through Minecraft Education Edition.^a^

Unit cost	Scenario A (€4500)^b^	Scenario B (€6000)
Per teacher (n=23)	€195.65	€260.87
Per project delivered or evaluated (n=27)	€166.67	€222.22
Per student (n=850)	€5.29	€7.06

a Costs are shown for 2 scenarios: implementation costs only (€4500) and total costs including awards (€6000).

b €1=US $1.15 as of August 3, 2026.

## Discussion

### Principal Findings

In this cross-sectional program evaluation, the Minecraft Education Edition CPR Museum Challenge demonstrated that a gamified, fully online, school-based format can achieve wide reach with relatively low provider-side costs while generating standardized, comparable learning artifacts that can be assessed at scale using a rubric. By enrolling 23 teachers and reaching 850 students within a 21-day window, the format may support scalable participation. These findings align with the European Resuscitation Council Guidelines 2025, which emphasize strengthening community readiness through innovative and accessible educational models [1,3]. Furthermore, the use of Minecraft Education Edition fits the endorsement of technology-enhanced learning to improve engagement, especially when traditional instructor-led training faces scaling barriers in schools, such as training of schoolteachers, confidence to teach, and/or physical training materials [12]. This also points out schools and teacher-facilitated models as a key public-health level for normalizing adult BLS education in early ages and encourages rigorous evaluation of school-appropriate educational strategies [12]. Finally, our results illustrate a pragmatic pathway to pairing educational innovation with structured measurement (participation, performance, and costs) that can inform future implementation and scale-up [11].

Despite the high engagement recorded, the substantial variability in rubric scores, ranging from 0 to 19, suggests that translating adult BLS concepts into a self-directed virtual adult BLS course remains a significant challenge for students. In terms of project characteristics, submitted projects tended to score higher in creativity and originality than in technical complexity. This highlights a known phenomenon in serious games, in which the ludic elements may overshadow the instructional goals when they are not carefully aligned with pedagogical intentions. In this case, playful features receive more attention than the education dimension, leading to games that are engaging but pedagogically weak or loosely connected to learning outcomes [25,26]. The low performance in “technology and mechanisms” indicates that technical barriers or a preference for narrative design might hinder the simulation of advanced functional elements. Most importantly, the deficit in BLS-specific domains reinforces the argument that digital materials must be rigorously updated and structured to ensure they translate into actual clinical competence [2]. While digital platforms like Minecraft offer a pragmatic pathway for scalability and enable skills to be refreshed [27], future iterations should incorporate instructional scaffolding, such as in-game nonplayer characters acting as mentors, to ensure that competence in lifesaving maneuvers is not lost in the fun. In the meantime, there is insufficient evidence to recommend specific game-based or app-based training methods over others [2,22,28].

The significant difference in performance across educational levels suggests that, while the initiative is inclusive, the current format favors the cognitive and technical maturity of secondary students. This is consistent with evidence showing that while children can recognize emergencies, the structured logic required for the Chain of Survival, the technical proficiency and physical strength required for high-quality CPR significantly improve after the age of 10 years [29-33]. For primary education, a more guided or “templated” approach is necessary to reduce the cognitive load while maintaining the engagement benefits of the platform.

One of the most compelling findings is the program’s cost efficiency. A total expenditure of €6000 (€7.06 per student) provides a pragmatic pathway for large-scale public health interventions, especially in low-resource settings. A key factor was the role of schoolteachers as facilitators; by integrating the challenge into the regular school schedule, the need for expensive health care personnel was removed. In addition, this model harnesses the pedagogical expertise of teachers, who possess the didactic skills necessary to adapt complex concepts to the specific developmental stages of the students [12,33-35]. Unlike external health care providers who may only offer a “one-off” session, schoolteachers might provide continuous reinforcement and longitudinal support, effectively integrating BLS into the broader school curriculum as a transversal competency [12,13,33-35]. However, for this model to be sustainable, adequate teacher training is paramount, as their self-efficacy directly influences student outcomes [34,35]. By shifting the leadership role to schoolteachers, this model may lower provider-side economic barriers and could contribute to a more sustainable community-wide resuscitation culture.

### Limitations and Future Lines of Work

This study has several limitations. First, it is a cross-sectional program evaluation of a single edition with voluntary participation, which limits causal inference and generalizability. Second, outcomes were based on rubric scoring of class-level project artifacts rather than objective measures of individual students’ adult BLS knowledge gain, psychomotor CPR quality, or real-world bystander behavior, and the wide dispersion of scores suggests heterogeneous uptake across submissions. Third, overlap between rubric domains and differences by educational level and video submission indicate potential confounding by maturity and communication skills. Finally, the cost analysis reflects a provider perspective without monetizing teacher time, which may not translate to other settings. Formal interrater reliability was not estimated because final rubric scores were assigned by consensus and preconsensus individual ratings were not retained.

Regarding statistical power, the 27 evaluated projects represent a census of all eligible submissions from a single edition rather than a random sample; an a priori power calculation for a target effect is therefore not applicable, and post hoc observed power would be uninformative. The inferential analyses should accordingly be interpreted as exploratory and hypothesis-generating, and the limited number of projects is reflected in the wide CIs reported. As a sensitivity consideration, with a sample size of 27 and a 2-sided α of .05, only large effects (Cliff delta on the order of 0.6) were detectable with adequate power, so smaller associations cannot be excluded. Moreover, the high intercorrelation among rubric domains (Spearman ρ up to 0.94; Cronbach α=0.95) indicates a single underlying construct rather than 4 independent variables, which is why multivariable modeling was not pursued.

Future work should strengthen scaffolding (especially in primary school), refine the rubric, and evaluate hybrid models combining Minecraft Education Edition with hands-on CPR training using controlled designs, pretesting or posttesting, and longer follow-up for retention and behavioral outcomes.

### Conclusions

In conclusion, this cross-sectional program evaluation suggests that, in this edition, a teacher-facilitated, fully online, gamified school initiative delivered through Minecraft Education Edition achieved wide reach at a relatively low provider cost while producing standardized class-level learning artifacts that could be assessed at scale using a structured rubric. However, the modest median performance and the marked dispersion of scores indicate that translating core adult BLS concepts into an in-game format remains challenging and may allow creativity to overshadow clinical precision. These findings primarily support the feasibility, reach, and structured assessment potential of the initiative rather than its educational effectiveness in terms of knowledge or skill acquisition. Future editions should therefore strengthen instructional scaffolding, particularly in primary education, and future controlled studies should assess knowledge and skill acquisition, retention, transfer to real-world bystander behavior, and the added value of hybrid models combining gamified learning with hands-on CPR practice and feedback. Digital gamified activities should be positioned as a complementary strategy for engagement, reinforcement, and refreshers, rather than as a substitute for practical CPR training.

## Supplementary material

10.2196/93141 Multimedia Appendix 1 Teachers’ guide.

10.2196/93141 Multimedia Appendix 2 Guía para el docente.

## Abbreviations

BLS: basic life support
CPR: cardiopulmonary resuscitation
UCAM: Universidad Católica de Murcia
