# Supplementary material for: Gamified Adult Basic Life Support Training for Schoolchildren Using Minecraft Education Edition: Cross-Sectional Program Evaluation
Source: JMIR Serious Games. 2026 Sep 9;14:e93141. doi: 10.2196/93141 (PMC13556288; doi:10.2196/93141)
Supplement: Supplementary file 1 [file games-v14-e93141-s001.pdf]

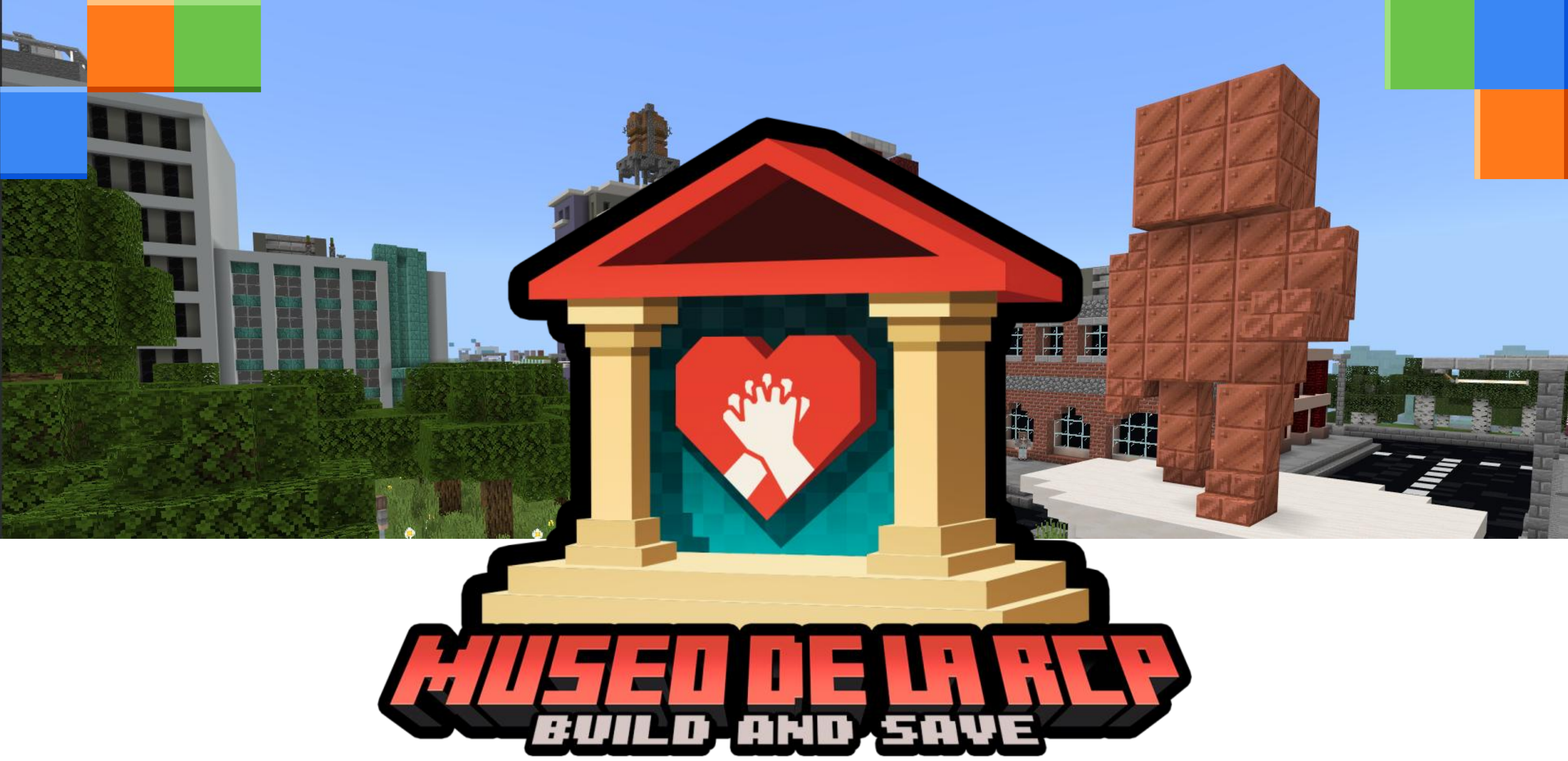

CHALLENGE GUIDE: Build & Save: CPR Museum

WORLD CARDIAC ARREST DAY

educacion@l3tcraft.com

Fundación MAPFRE   UCAM RCP Proyecto de divulgación   L3TCRAFT EDUCACIÓN

World Cardiac Arrest Day – October 16

The Cardiac Arrest Awareness Day was created in 2012, thanks to the Declaration of the European Parliament to establish a European Cardiac Arrest Awareness Week.

It takes place on October 16 every year and, since 2018, it has been celebrated worldwide, thanks to the support of ILCOR (International Liaison Committee on Resuscitation), which brings together the Cardiopulmonary Resuscitation Councils from the 5 continents.

With this challenge, the goal is to raise awareness about the importance of Cardiopulmonary Resuscitation (CPR) by **building a CPR Museum in Minecraft** with rooms that represent the basic steps: check, call for help, compress, and defibrillate.

🖼️ Build & Save: CPR Museum

Challenge Objectives:

- Understand what CPR (Cardiopulmonary Resuscitation) means.
- Raise awareness about the importance of being trained and prepared to help in an emergency.
- Represent in Minecraft a space that highlights the importance of raising awareness in society about saving lives.

Before building, it is recommended that students conduct **research** on topics related to cardiac arrest or the importance of health and acting quickly in these situations.

You have several **resources** to find information about these topics, which you can consult below:

- https://www.cercp.org/dia-mundial-de-la-parada-cardiaca/

- https://www.fundacionmapfre.org/educacion-divulgacion/salud-bienestar/aprendiendo-juntos-salvar-vidas/recursos-apoyo/

For this activity, students will use Minecraft Education to develop their building proposals. You can download the tool directly here. If you don't have licenses, you can write to us at educacion@l3tcraft.com to request them.

Explanation of the basic steps for the museum rooms

🔎 Room 1: Check

- **Objective:** Represent the first action: check if the person responds.
- **What it should include:**
    - A figure/character lying on the ground.
    - Another character (NPC) approaching or looking, symbolizing the action of checking.
    - Signs with phrases like: "Are you okay?" or "Check if they respond." Recommendations that would be given for the checking phase.

Examples in Minecraft:
A doll made of wool or red clay lying down. NPCs can also be placed with conversations simulating a "check" situation. A pixel art heart that is turned off or broken to symbolize the emergency.

📞 Room 2: Call for Help

- **Objective:** Show that it is necessary to call emergency services (112).
- **What it should include:**
    - A giant pixel art phone.
    - NPC delivering the message: "Call 112 or ask someone nearby for help."
    - Symbols of megaphones, bells, or even a sign with the emergency number.

Examples in Minecraft:
A mobile phone built with black and gray concrete blocks, with red numbers. Signs with messages like: "Ask for help quickly!" or phone information or what details they need to provide when making the call. Characters representing family, friends, or healthcare professionals responding.

🙌 Room 3: Compress

- **Objective:** Represent chest compressions.
- **What it should include:**
    - Two giant hands made with blocks, in a pressing position on a chest or heart.
    - A practice dummy (figure made of wool or terracotta).
    - Signs with messages like: "30 continuous compressions" or "Push with straight arms."
    - NPCs with external links to videos on how to perform compressions.

Examples in Minecraft:
Pixel art of two hands on a heart. Redstone to simulate movement (pistons moving up and down). NPC that says: "Push hard and fast in the center of the chest" and provides external links as resources.

Explanation of the basic steps for the museum rooms

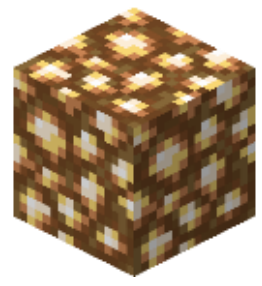

Room 4: Defibrillate (AED)

- **Objective:** Raise awareness about the use of the defibrillator (AED).
- **What it should include:**
    - A giant AED (green box with a yellow lightning bolt).
    - Pixel art heart illuminated with glowstone that lights up when the AED is "activated."
    - Signs with messages like: "Follow the AED instructions" or "Apply the pads and wait for the shock."

Examples in Minecraft:
Pixel art of a defibrillator with lightning. Use of redstone to light up lamps when activating a lever to simulate the use of a defibrillator. NPC that explains: "The AED will tell you step by step what to do" and gives guidance until healthcare professionals arrive.

Extra: Final Room / Reflection Space

An extra room can be added inside the museum as a final reflection space, or students can add something additional to the CPR steps. For example: **a giant heart beating with some redstone mechanism** to represent life recovered thanks to following the CPR steps.

Closing messages: "Anyone can be a hero if they know CPR."
Area for group photos or virtual signatures from the teams.

Redstone is like **electricity** inside Minecraft: it is **red dust** (looks like a mineral) that you place on the ground to create **circuits**.

These circuits allow some things in the game to activate others: for example, a button turning on a lamp, a lever opening a door, or a mechanism moving.

Simple classroom examples:

- Place a **Redstone block** and connect it to a lamp to make it light up.
- Use a **lever** that, when activated, opens a door.
- Make a **piston** go up and down as if it were an elevator or to simulate a "heartbeat" in a build.

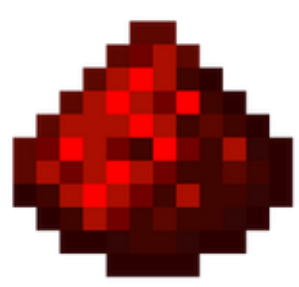

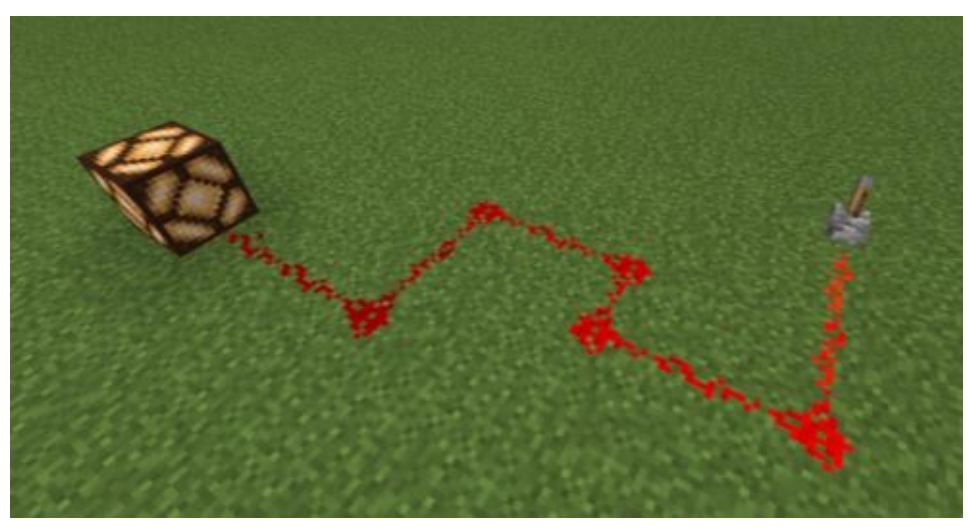

Instructions

Below is a possible schedule for the activity to participate in the World Cardiac Arrest Day Challenge.

Step 1: Research World Cardiac Arrest Day

Teachers can work with groups of students in the same class. Each group will work on their proposal for the CPR museum. They can decorate and design the museum freely, inspired by a museum they know or by inventing and creating one from scratch.

Before starting to build, they can carry out an initial research phase to plan how to begin creating the museum in Minecraft. In this research phase, they can make sketches, drawings, and reflect on how to represent the basic CPR steps: check, call for help, compress, and defibrillate.

Step 2: Build their CPR Minecraft Museum

When carrying out the construction in the game, the following aspects should be taken into account:

- Students must play in Creative Mode to have all building items unlocked.
- Students must document their work using the book & quill and the camera. If they wish, they can document their work outside Minecraft, with screenshots or photos.
- When creating the world where the construction will take place:
    - A flat world can be created to start building like a blank canvas.
    - The CPR world provided on the contest website can be used, with the dedicated construction space.

Step 3: Export and submit the completed work

Once the construction is finished, it is important to save the world exactly at the point where the work should be evaluated. In other words, students must prepare the world and other files, ready to send for evaluation. The submission section will contain all the requirements for sending the world.

The final submission must be made by the TEACHER, sending up to one project per class participating in the competition.

Group Organization

The entire class

The teacher may propose that the whole class participate in building the project, always considering the limitations in terms of connection and number of simultaneous users. Minecraft Education allows up to a maximum of 40 users playing in the same world. It must be taken into account that the larger the number of users in the same world, the more demanding the game will be in terms of device and connection.

Small groups

The teacher may choose to work on the topic with small groups within the same classroom. We recommend a maximum of 5 members per group. Each group can propose a different project idea or divide the roles and tasks related to the single project they will create.

⚠️ Only **one project** may be submitted per class. Therefore, if a class has worked on different proposals with small groups, the teacher and the class will have to choose which proposal to submit (this can be done through an internal vote).

If your class is working in several groups to develop different building projects, you can use the evaluation rubric yourselves, and the students, to vote on the project that will represent the class, allowing them to take part in the decision.

If you are working with a course, for example, for 2º ESO, you may submit one project per class, meaning one project for 2º ESO A, another for 2º ESO B, etc.

Deadlines and Submissions

The deadline to submit projects is **October 13, 2025, at 23:59**. Projects may be submitted before that date.

Registrations for the Challenge will remain open from October 22. Starting October 13, the evaluation of projects will take place, and the date of the final event will be announced, which will be held on October 16 to showcase the best projects and celebrate World Cardiac Arrest Day.

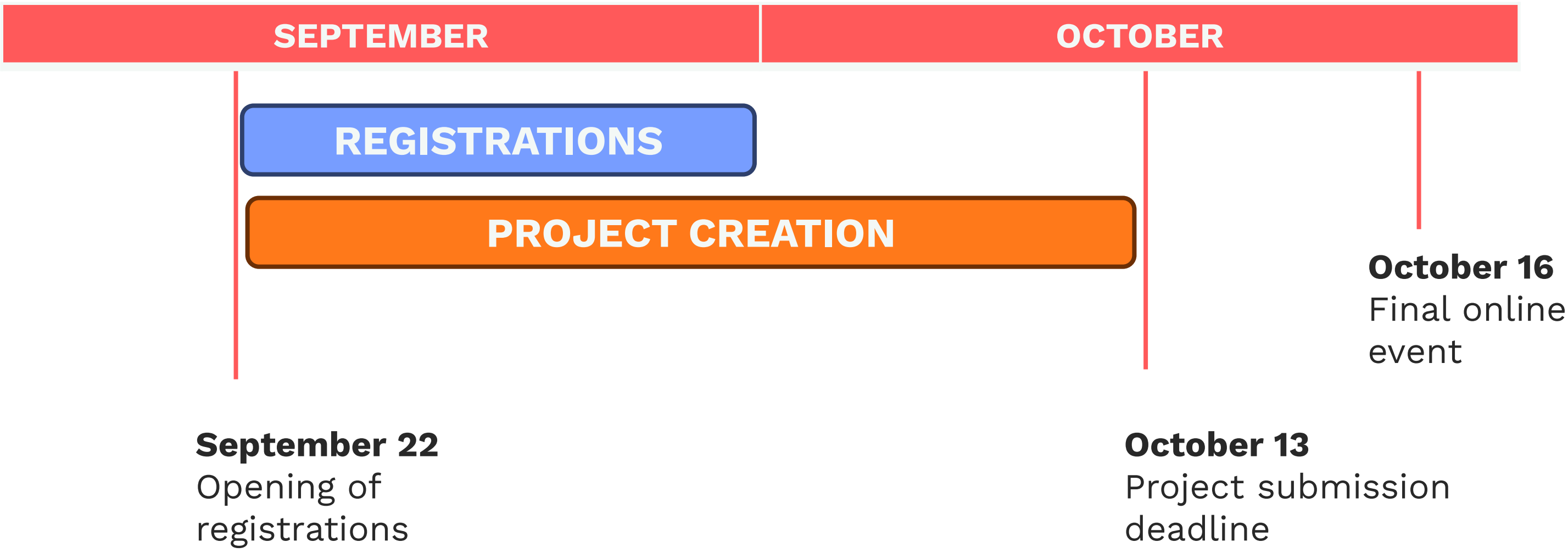

For the Challenge jury to evaluate the work, the teacher in charge of each team must submit the following evidence:

1. **Project submission form:** this must be completed once for each project submitted. For example, if a teacher submits 3 projects, they must fill out the form 3 times, once for each project.
2. When submitting the work, the teacher must have the following evidence prepared:
    - **a)** **.pdf file** corresponding to the work diary created by the students, including photos and explanations.
    - **b)** **.mcworld file**, corresponding to the developed project/world. This file must be stored in the cloud and submitted via a public view link so it can be downloaded and evaluated by the jury.

Optional:

- **Explanatory video of the project**, with images showing the build and a corresponding description, no longer than 2 minutes.

Submission Method

The teacher must submit the projects through the project submission form **https://bit.ly/CPRDeliveryProjects2025**. Remember that you can assign a name to your project along with the group of students who created it, referring to the theme of CPR Museum 2025. This name must be included in the project submission.

The steps to follow before submission are as follows:

Step 1:

Create a folder in a cloud location such as Google Drive or OneDrive and make sure the permissions of this folder are set to “PUBLIC,” meaning that “anyone” can view the content.

Step 2:

In this folder, include the explanatory PDF of the project, which may be the book & quill with screenshots taken using the in-game camera, created by the students about the construction of their CPR museum. You may also add the optional project explanation video to this folder.

Step 3:

Export the Minecraft world in .mcworld format. When exporting the world, make sure to create a /setworldspawn (with the coordinates) or save the world and then export it at the point where you want the project evaluation to begin. Once exported, save it in the corresponding folder you created with public view permissions.

Step 4:

Collect the public links of each of the created resources and complete the LEG 2025 project submission form through the following link https://bit.ly/CPRDeliveryProjects2025.

⚠️ Do not forget that if you participate with several classes, you must fill out **ONE FORM PER PROJECT/CLASS** submitted. Also remember to organize the shared folders with public visibility according to this requirement.

Evaluation Rubric

The Challenge jury will use the following rubric to evaluate all participating projects:

	1	2	3	4
**Creativity and originality**	The construction is basic and follows a common design.	The construction shows some creativity but remains simple.	The construction is creative and has original elements.	The construction is very creative and unique, with many original elements.
**Technology and mechanisms (redstone, command blocks...)**	No advanced technologies are used.	Some basic technologies and mechanisms are used.	Several advanced technologies and mechanisms are used.	Many advanced technologies and mechanisms are used, including innovative automation systems.
**Consistency with the theme (CPR rooms)**	Weak or non-existent reflection of CPR steps.	Reflection present but not entirely clear with the CPR steps.	Reflection present and clearly shown for some CPR steps.	Each room clearly reflects a CPR step and its importance (symbols, NPCs, explained processes, etc.).
**Integration of content in the CPR museum**	The world contains no content elements such as boards or NPCs.	The world contains some content such as boards or NPCs.	The world contains several types of content such as boards or NPCs.	The world contains many types of content such as boards or NPCs.
**Explanation of content in the world**	The world explanation is poor and confusing.	The explanation within the world is acceptable and clear in some aspects.	The explanation of the world's elements is good and clear, with some coherence between items such as boards and NPCs.	The explanation of the world's elements is excellent, very clear and detailed, with high coherence between items such as boards and NPCs.

📹 An **extra +1 point** will be added to the final score if the submitted project includes an explanatory video of the project (recommended length **under 2 minutes**).

Explanation of the rubric and items to be evaluated

Creativity and originality

This item evaluates the ability to design and build museums that are unique and original. Creativity in design, incorporation of innovative elements, and the ability to think outside the box will be valued. Students must demonstrate that they have used their imagination to create something not only functional but also aesthetically attractive and different from the conventional, with clear messages about the importance of knowing the CPR steps to save lives.

Technology and mechanisms (redstone, command blocks...)

This item focuses on the innovative aspect of the Museum. It will assess how technologies and mechanisms have been used to improve the usability and efficiency of the Museum. For example, the use of redstone to create automated systems such as automatic doors, lighting and security systems, or the use of solar panels to make the museum more sustainable.

Consistency with the theme (CPR rooms)

Here the **integration** of the different **steps** to follow in a **cardiac arrest** situation will be **evaluated**, and these will be represented in the Museum rooms built by the students. It is important that each of the rooms can be clearly identified with the step they represent, as well as the creativity in integrating elements that represent each of the situations.

Integration of content in the CPR museum

This item evaluates the **amount of content elements** that students have used to make the importance of Cardiopulmonary Resuscitation (CPR) clear. To achieve the highest score in this rubric item, it will be necessary to see a significant number of boards and NPCs in the museum that support the explanation of content across the different rooms of the basic CPR steps: check, call for help, compress, and defibrillate.

Explanation of content in the world

This item evaluates the students' ability to **explain and justify their construction** within the Minecraft world. **Clear and detailed descriptions** of the museum elements must be provided, using items such as boards and NPCs to guide visitors. Clarity and organization of information will be valued, as well as the ability to communicate and explain their creations.

Examples of worlds in Minecraft Education

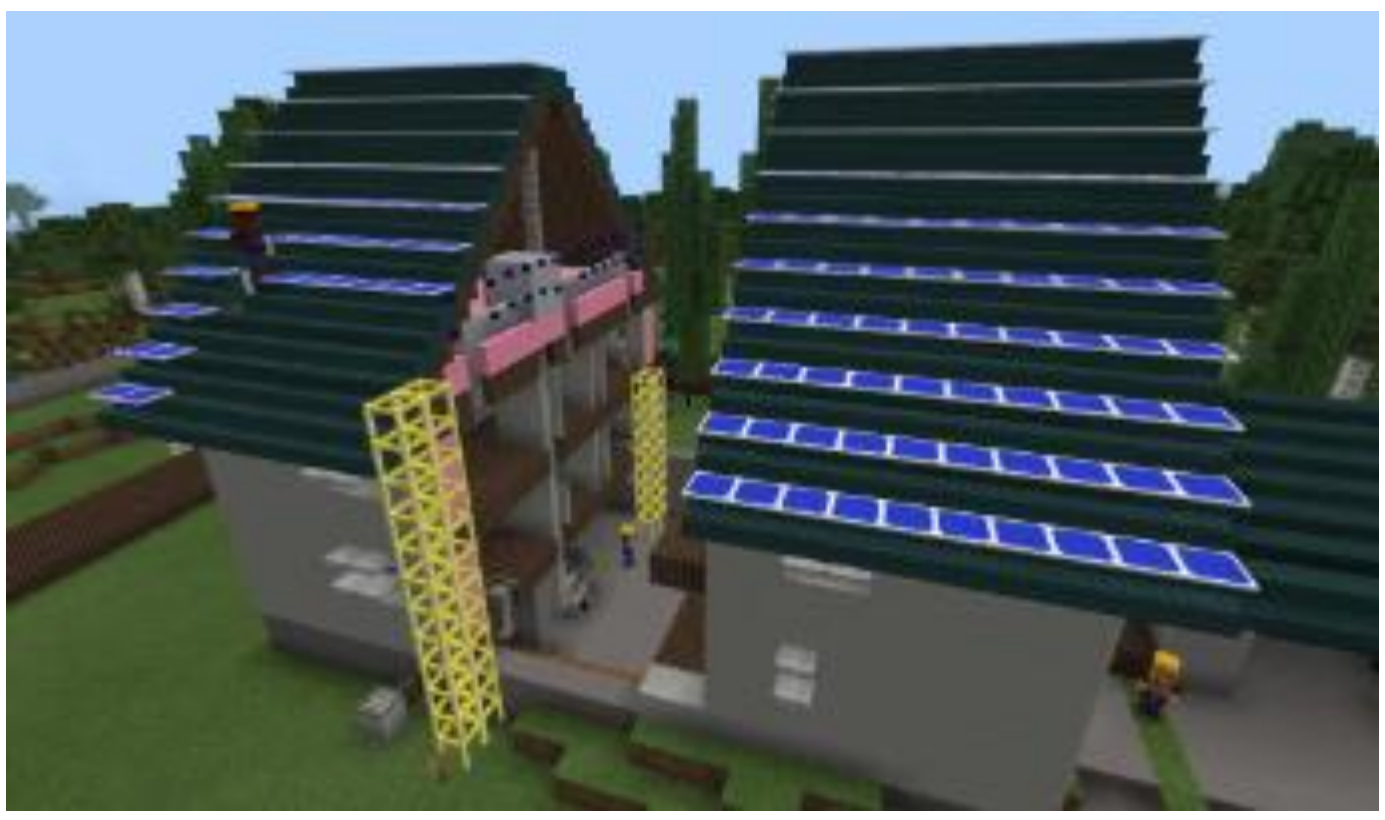

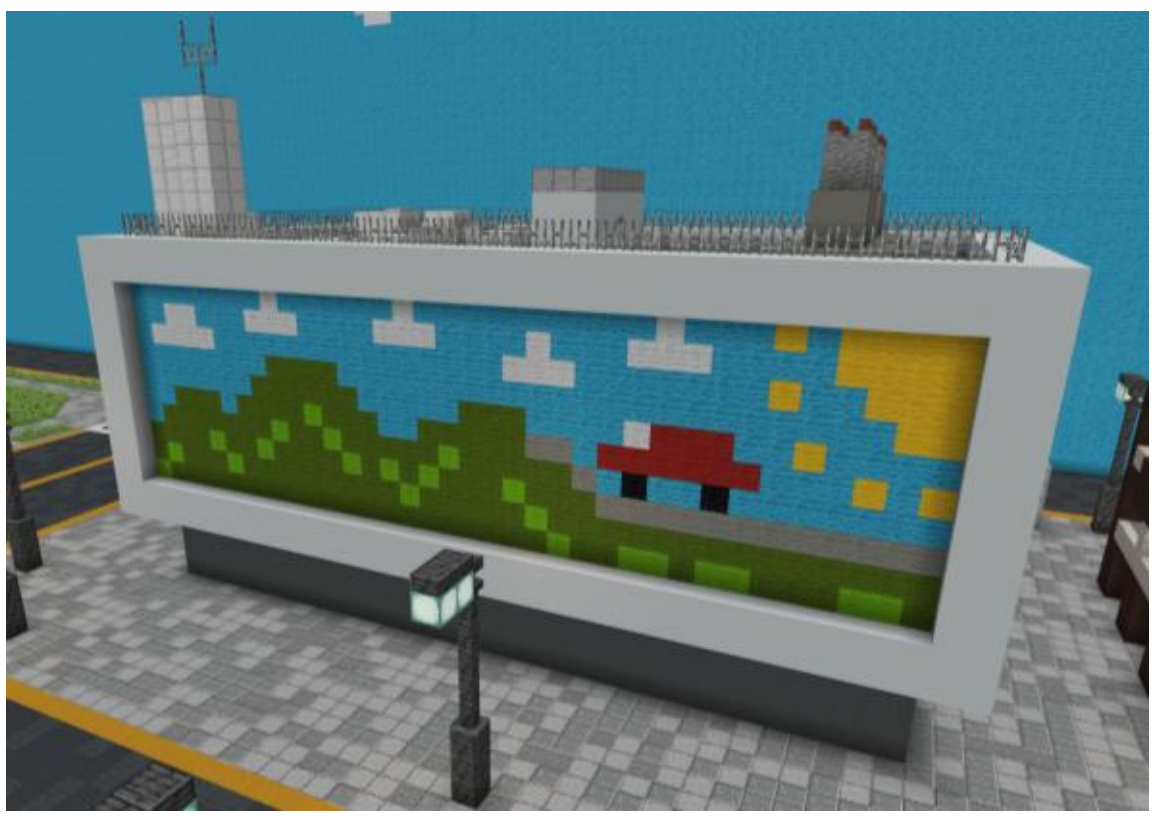

Prizes

The top 3 projects will receive exclusive prizes. On Letcraft Educación social media, we will be announcing the best-ranked projects, and during the online event taking place on October 16, we will reveal the winners.

All teachers and students who participate will receive a certificate of participation in the World Cardiac Arrest Day 2025 Challenge.

First prize: 500€ in school supplies

Second prize: 500€ in school supplies

Third prize: 500€ in school supplies

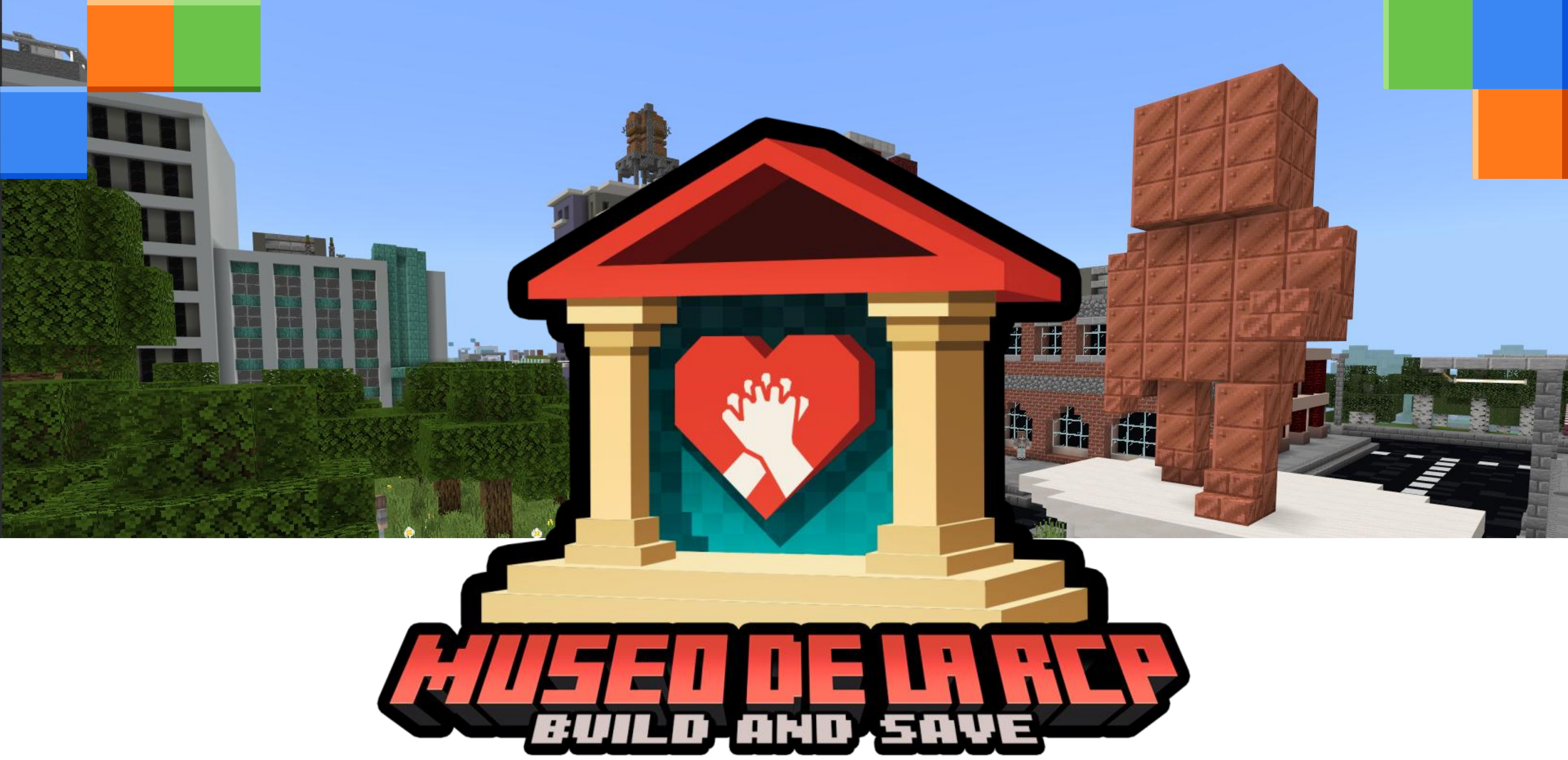

Thank you! ;)

Contact

educacion@l3tcraft.com

https://bit.ly/CPRMinecraft

Fundación MAPFRE

UCAM RCP Proyecto de divulgación

L3TCRAFT EDUCACIÓN
