# Supplementary material for: Gamified Adult Basic Life Support Training for Schoolchildren Using Minecraft Education Edition: Cross-Sectional Program Evaluation
Source: JMIR Serious Games. 2026 Sep 9;14:e93141. doi: 10.2196/93141 (PMC13556288; doi:10.2196/93141)
Supplement: Supplementary file 2 [file games-v14-e93141-s002.pdf]

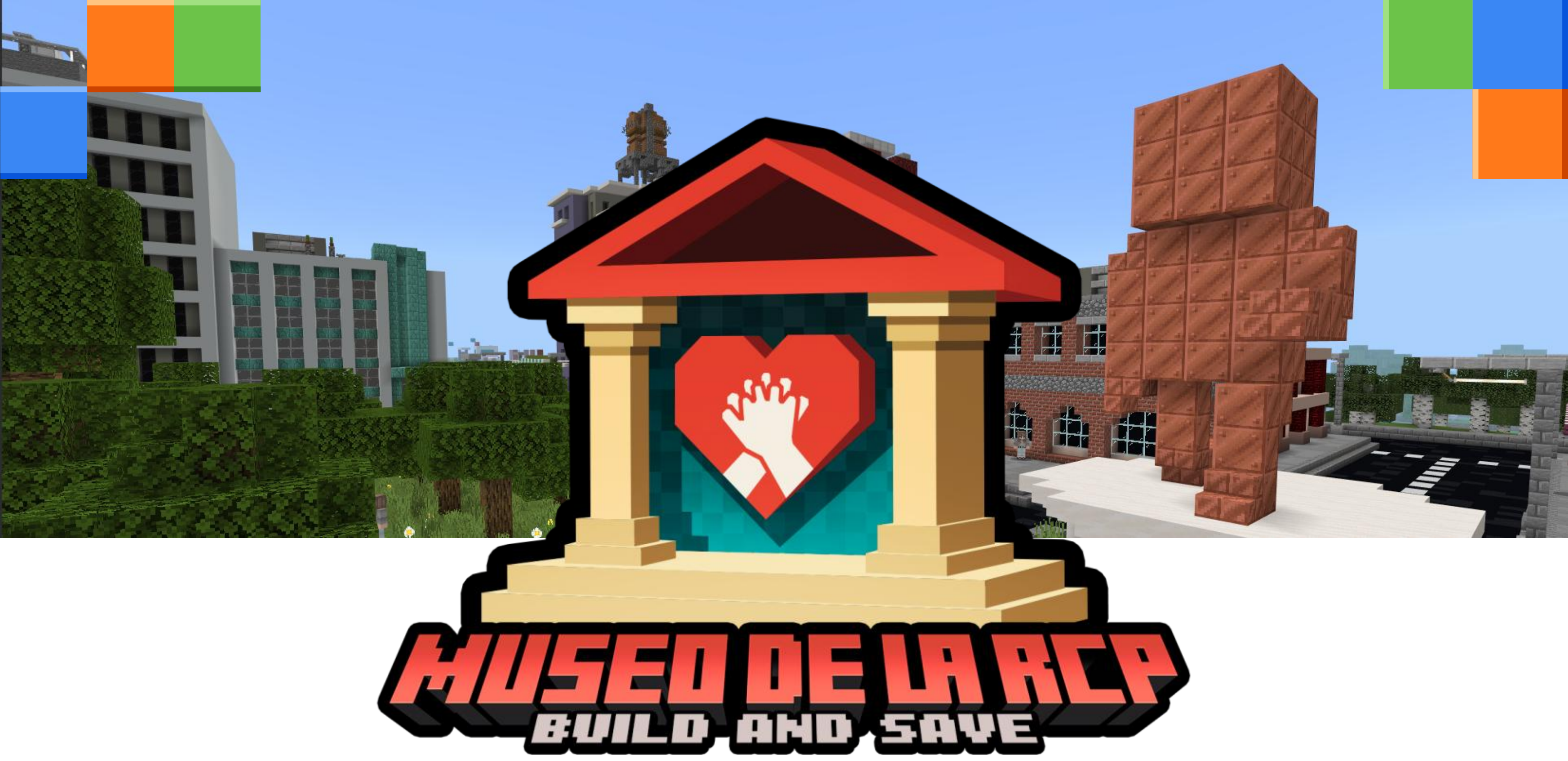

GUÍA DEL CHALLENGE: Build & Save: Museo de la RCP

DÍA MUNDIA DE LA PARADA CARDIACA

educacion@l3tcraft.com

Fundación MAPFRE

UCAM RCP Proyecto de divulgación

L3TCRAFT EDUCACIÓN

Día Mundial de la Parada Cardiaca – 16 octubre

El Día de concienciación ante la parada cardiaca fue creado en el año 2012, gracias a la Declaración del Parlamento Europeo para crear una semana europea de concienciación ante la parada cardiaca.

Tiene lugar el 16 de octubre de cada año y, desde el año 2018, se celebra a nivel mundial, gracias al apoyo del ILCOR (International Liaison Committee on Resuscitation), que agrupa a los Consejos de Resucitación Cardiopulmonar de los 5 continentes.

Con este challenge se buscará concienciar sobre la importancia de la Reanimación Cardiopulmonar (RCP) construyendo en Minecraft un **Museo de la RCP con salas que representen los pasos básicos**: comprobar, pedir ayuda, comprimir y desfibrilar.

🖼️ Build & Save: Museo de la RCP

Objetivos del challenge:

- Comprender qué significa RCP (Reanimación Cardiopulmonar).
- Concienciar sobre la importancia de estar formados y preparados para ayudar en una emergencia.
- Representar en Minecraft un espacio con el que recojan la importancia de sensibilizar a la sociedad sobre salvar vidas.

Previamente a la construcción, se aconseja que los estudiantes realicen una **investigación** acerca de los contenidos relacionados con las paradas cardiacas o la importancia de la salud y de actuar rápido en estas situaciones.

Tenéis varios **recursos** para encontrar información acerca de estos contenidos que podéis consultar a continuación:

- https://www.cercp.org/dia-mundial-de-la-parada-cardiaca/

- https://www.fundacionmapfre.org/educacion-divulgacion/salud-bienestar/aprendiendo-juntos-salvar-vidas/recursos-apoyo/

Para la realización de esta actividad, los estudiantes utilizarán Minecraft Education para el desarrollo de sus propuestas de construcción. Puedes descargar la herramienta directamente aquí. Si no dispones de licencias, puedes escribirnos a educacion@l3tcraft.com para solicitarlas.

Explicación de los pasos básicos para las salas del museo

🔎 **Sala 1: Comprobar**

- **Objetivo:** Representar la primera acción: comprobar si la persona responde.
- **Qué debe tener:**
  - Una figura/personaje tumbado en el suelo.
  - Otro personaje (NPC) que se acerque o mire, simbolizando la acción de comprobar.
  - Carteles con frases como: *"¿Estás bien?"* o *"Comprueba si responde"*. Recomendaciones que se darían para la fase de comprobación.

Ejemplos en Minecraft:
Un muñeco hecho con lana o arcilla roja tumbado. También se pueden colocar personajes NPC con conversaciones que simulen una situación de "comprueba". Un corazón pixel art que esté apagado o roto para simbolizar la emergencia.

📞 **Sala 2: Pedir ayuda**

- **Objetivo:** Mostrar que hay que avisar a emergencias (112).
- **Qué debe tener:**
  - Un teléfono gigante en pixel art.
  - NPC que dé el mensaje: *"Llama al 112 o pide ayuda a alguien cercano"*.
  - Símbolos de megáfonos, campanas o incluso un cartel con el número de emergencias.

Ejemplos en Minecraft:
Un teléfono móvil construido con bloques de concreto negro y gris, con números rojos. Pizarras con mensajes tipo: *"¡Pide ayuda rápido!"* o información del teléfono o qué información tienen que dar cuando hacen la llamada de pedir ayuda. Personajes representando familiares, amigos o sanitarios que responden.

🙌 **Sala 3: Comprimir**

- **Objetivo:** Representar las compresiones torácicas.
- **Qué debe tener:**
  - Dos manos gigantes hechas con bloques, en posición de presión sobre un pecho o corazón.
  - Un muñeco de práctica (figura de lana o terracota).
  - Carteles con mensajes como: *"30 compresiones seguidas"* o *"Haz fuerza con los brazos rectos"*.
  - NPCs con enlaces externos a vídeos de cómo realizar las compresiones.

Ejemplos en Minecraft:
Pixel art de dos manos sobre un corazón. Redstone para simular un movimiento (pistones subiendo y bajando). NPC que diga: *"Comprime fuerte y rápido en el centro del pecho"* y que tenga enlaces externos como recursos.

Explicación de los pasos básicos para las salas del museo

Sala 4: Desfibrilar (DEA)

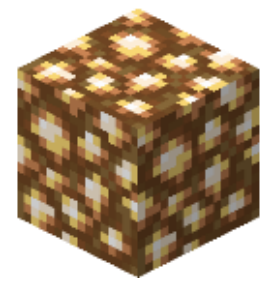

- **Objetivo:** Concienciar sobre el uso del desfibrilador (DEA).
- **Qué debe tener:**
    - Un DEA gigante (caja verde con rayo amarillo).
    - Corazón pixel art iluminado con glowstone que se enciende al "activar" el DEA.
    - Carteles con mensajes: *"Sigue las instrucciones del DEA"* o *"Aplica los parches y espera la descarga"*.

Ejemplos en Minecraft:
Pixel art de un desfibrilador con rayos. Uso de redstone para encender lámparas al activar una palanca y simular el uso de un desfibrilador. NPC que explique: *"El DEA te dirá qué hacer paso a paso"* y pautas hasta que lleguen los profesionales sanitarios.

Extra: Sala final / Espacio de reflexión

Se puede plantear alguna sala extra dentro del museo que sea un espacio donde los estudiantes construyan un espacio de reflexión final o añadan algo extra a los pasos de la RCP. Por ejemplo: un **corazón gigante latiendo con algún mecanismo de redstone** que represente la vida recuperada gracias a seguir los pasos de la RCP.

Mensajes de cierre: *"Cualquiera puede ser un héroe si sabe RCP"*.
Zona para fotos de grupo o firmas virtuales de los equipos.

El **Redstone** es como la **electricidad** dentro de Minecraft: son **polvos rojos** (parecen como un mineral) que se colocan en el suelo para crear **circuitos**.

Estos circuitos permiten que unas cosas en el juego activen otras: por ejemplo, que un botón encienda una lámpara, que una palanca abra una puerta o que un mecanismo se mueva.

Ejemplos sencillos de uso en clase:
- Colocar un **bloque de Redstone** y conectarlo a una lámpara para que se encienda.
- Usar una **palanca** que, al activarse, abra una puerta.
- Hacer que un **pistón** suba y baje como si fuese un ascensor o para simular un "latido de corazón" en una construcción.

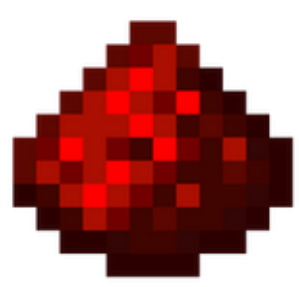

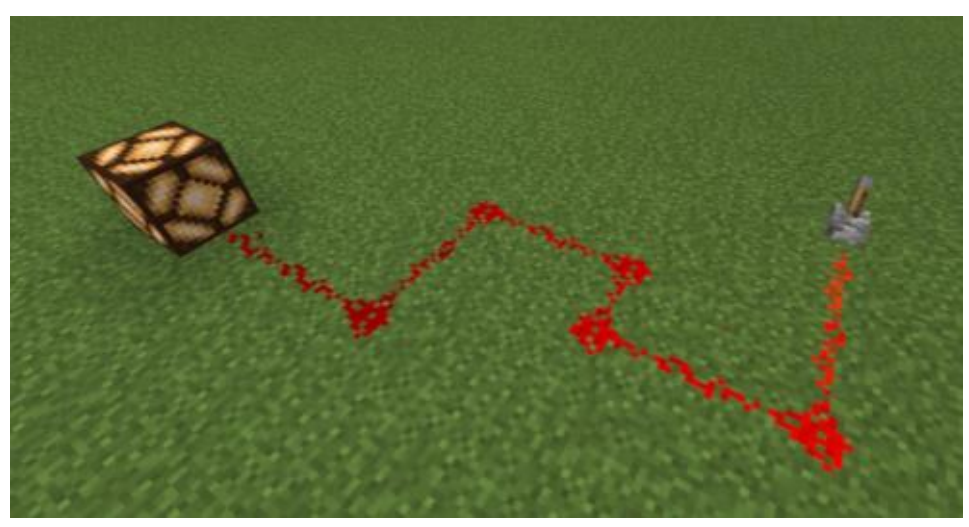

Instrucciones

A continuación, se presenta una posible programación de la actividad para participar en el Challenge del Día Mundial de la Parada Cardiaca

Paso 1: Investigar sobre el Día Mundial de la Parada Cardiaca

Los docentes podrán trabajar por grupos de estudiantes en una misma clase. Cada grupo deberá trabajar en su propuesta museo de la RCP. Pueden decorar y ambientar el museo libremente, inspirándose en un museo que ellos conozcan o inventándose y creando uno desde cero.

Antes de ponerse a construir, pueden realizar una primera fase de investigación para planificar cómo empezar a crear el museo en Miencraft. En la fase de investigación pueden crear planos, dibujos, reflexionar sobre cómo pueden representar los pasos básicos de la RCP: comprobar, pedir ayuda, comprimir y desfibrilar.

Paso 2: Construir su museo RCP Minecraft

A la hora de realizar la construcción en el juego, debemos tener en cuenta los siguientes aspectos:

- Los estudiantes deben jugar en modo de juego creativo, para poder tener todos los ítems de construcción desbloqueados.
- Los estudiantes deben documentar su trabajo a través del libro y la pluma y la cámara. Si quieren, pueden documentar su trabajo fuera de Minecraft, con capturas o fotos.
- A la hora de crear el mundo en el que se lleve a cabo la construcción:
    - Se puede crear un mundo plano, para empezar a construir como un lienzo en blanco.
    - Se puede utilizar el mundo RCP que encontrarán en la web del concurso y utilizar el espacio de construcción creado para ello.

Paso 3: Exportar y enviar el trabajo realizado

Una vez completada la construcción, será importante guardar el mundo exactamente en el punto donde queremos que se evalúe el trabajo, es decir, los estudiantes deberán preparar el mundo y otros archivos, listos para enviar a evaluar. En el apartado de entregas se podrá consultar todos los requisitos de envío del mundo.

La entrega final deberá realizarla el DOCENTE, enviando hasta un máximo de un proyecto por clase participante en la competición.

Organización del grupo

La clase al completo

El docente puede proponer que toda la clase participe en el proceso de construcción de la propuesta, siempre teniendo en cuenta las limitaciones en cuanto a conexión y número de usuarios simultáneos. Minecraft Education permite hasta un máximo de 40 usuarios jugando en un mismo mundo. Habrá que tener en cuenta que cuanto mayor sea el número de usuarios dentro de un mismo mundo, más demandante será el juego en cuanto a dispositivo y conexión.

Grupos reducidos

El docente podrá optar por trabajar la temática con grupos reducidos dentro de una misma aula. Recomendamos un máximo de 5 integrantes por grupo. Cada grupo puede plantear una propuesta de proyecto distinta o repartirse los roles y trabajos relacionados con la propuesta única que vayan a crear.

⚠️ Sólo se podrá entregar **un proyecto** por clase, por lo que, si una misma clase ha trabajado en diferentes propuestas con grupos reducidos, el docente con su clase tendrá que elegir qué propuesta enviar (se puede hacer por votación interna).

Si en tu clase vais a hacer varios grupos para desarrollar varios proyectos de construcción, podéis utilizar la rúbrica de evaluación vosotros mismos y los estudiantes para votar el proyecto que representaría a su clase y que sean ellos los partícipes de la decisión.

Si estás trabajando con un curso, por ejemplo, de 2º ESO, podrás entregar un proyecto por cada clase, es decir, un proyecto por 2º ESO A, otro para 2º ESO B, etc.

Plazos y entregas

El último día para entregar los proyectos será el **13 de octubre de 2025 a las 23:59 h.** Los proyectos podrán entregarse antes de esa fecha.

Las inscripciones al Challenge permanecerán abiertas desde el 22 de octubre. A partir del 13 de octubre se llevará a cabo la evaluación de proyectos y se anunciará la fecha del evento final que tendrá lugar el 16 de octubre para conocer los mejores proyectos y celebrar el Día Mundial de la Parada Cardiaca

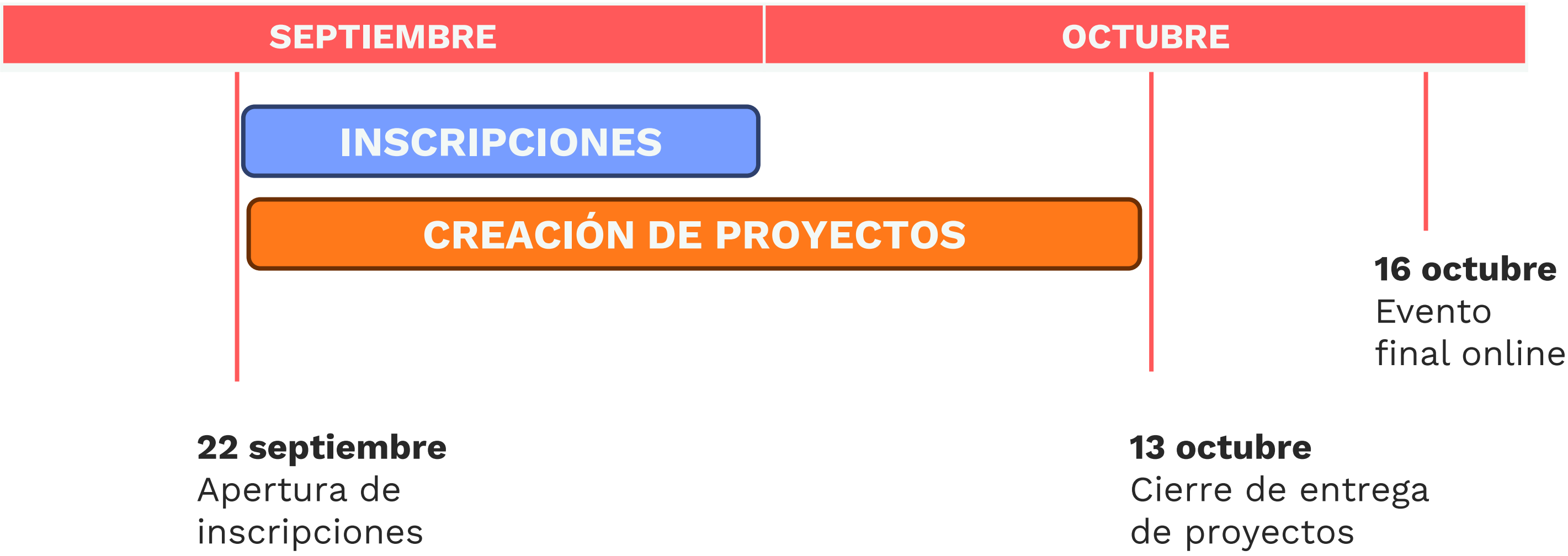

Para que el jurado del Challenge pueda evaluar el trabajo realizado, el docente a cargo de cada uno de los equipos deberá enviar las siguientes evidencias:

1. **Formulario de envío de proyecto**: este se rellenará una vez por cada proyecto enviado, es decir, si un docente presenta 3 proyectos, deberá rellenar el formulario de entrega 3 veces, una vez por cada proyecto.
2. Al enviar los trabajos el docente deberá tener preparadas las siguientes evidencias:
   - **a) Archivo en .pdf** correspondiente al diario de trabajo que han creado los estudiantes, con fotografías y explicaciones.
   - **b) Archivo en .mcworld**, correspondiente al proyecto/mundo desarrollado. Este archivo deberá ubicarse en la nube para poder enviarlo a través de un enlace con vista pública, para poder descargarse y evaluarse por parte del jurado.

Opcional:

- **Vídeo explicativo del proyecto**, con imágenes que permitan apreciar la construcción y su correspondiente descripción, no más de 2 minutos de duración.

Fundación MAPFRE · UCAM RCP Proyecto de divulgación · LETCRAFT EDUCACIÓN

Método de entrega

El docente deberá realizar las entregas a través del formulario de entrega de proyectos **https://bit.ly/EntregaProyectosRCPChallenge2025**. Recuerda que puedes asignar un nombre a tu proyecto junto con el grupo de estudiantes que lo han creado, en referencia a la temática de Museo RCP 2025. Este nombre deberás incluirlo en la entrega de proyectos.

Los pasos para seguir antes de realizar la entrega son los siguientes:

Paso 1:

Crea una carpeta en una ubicación en la nube como Google Drive o One Drive y asegúrate de que los permisos de esta carpeta están configurados como "PÚBLICOS", es decir, que puedan ver el contenido "todo el mundo".

Paso 2:

En esta carpeta incluye el PDF explicativo del proyecto, que puede ser el libro y la pluma con las capturas realizadas con la cámara dentro del juego, y que han generado los estudiantes sobre la construcción de su museo RCP. También puedes incorporar en esta carpeta el vídeo opcional de explicación del proyecto.

Paso 3:

Exporta el mundo de Minecraft en formato .mcworld. Ten en cuenta que a la hora de exportar el mundo deberás crear un /setworldspawn (con las coordenadas) o bien guardar el mundo y luego exportarlo en el punto en el que quieras que comience la evaluación del proyecto. Una vez exportado, podrás guardarlo en la carpeta correspondiente que has creado con permisos de visualización.

Paso 4:

Recopila los enlaces públicos de cada uno de los recursos creados y rellena el formulario de entrega de proyecto de LEG 2025 a través del siguiente enlace https://bit.ly/EntregaProyectosRCPChallenge2025.

⚠️ No olvides que si participas con varias clases deberás rellenar **UN FORMULARIO POR PROYECTO/CLASE** presentado. Recuerda también organizar las carpetas compartidas con visibilidad pública atendiendo a este requerimiento.

Rúbrica de evaluación

El jurado del Challenge utilizará la siguiente rúbrica para valorar todos los proyectos que participen:

	1	2	3	4
**Creatividad y originalidad**	La construcción es básica y sigue un diseño común.	La construcción muestra algo de creatividad, pero sigue siendo simple.	La construcción es creativa y tiene elementos originales.	La construcción es muy creativa y única, con muchos elementos originales.
**Tecnología y mecanismos (redstone, bloques de comando...)**	No se utilizan tecnologías avanzadas.	Se utilizan algunas tecnologías y mecanismos básicos.	Se utilizan varias tecnologías y mecanismos avanzados.	Se utilizan muchas tecnologías y mecanismos avanzados y sistemas de automatización innovadores.
**Coherencia con el tema (salas RCP)**	Reflejo débil o inexistente con los pasos de la RCP.	Reflejo presente pero no del todo clara con los pasos de la RCP.	Reflejo presente y con claridad sobre algunos de los pasos de la RCP.	Cada sala refleja claramente un paso de la RCP y su importancia (símbolos, NPC, procesos explicados, etc.).
**Integración de los contenidos en el museo RCP**	El mundo no contiene ningún tipo de contenido como pizarras o NPCs.	El mundo contiene algún tipo de contenido en pizarras o NPCs.	El mundo contiene varios tipos de contenido en pizarras o NPCs.	El mundo contiene muchos tipos de contenido en pizarras o NPCs.
**Explicación de los contenidos en el mundo**	La explicación del mundo es pobre y confusa.	La explicación dentro del mundo es aceptable y clara en algunos aspectos.	La explicación de los elementos del mundo es buena y clara, hay cierta coherencia entre cada uno de los ítems como pizarras y NPCs.	La explicación de los elementos del mundo es excelente, muy clara y detallada y tiene una coherencia alta entre los ítems colocados como pizarras y NPCs.

📹 Se otorgará **+1 punto extra** a la puntuación final si el proyecto a entregar incluye un vídeo explicativo del proyecto (recomendación **menos de 2 minutos**).

Fundación MAPFRE · UCAM RCP Proyecto de divulgación · LETCRAFT EDUCACIÓN

Explicación de la rúbrica e ítems a evaluar

Creatividad y originalidad

Este ítem evalúa la capacidad para diseñar y construir museos que sean únicas y originales. Se valorará la creatividad en el diseño, la incorporación de elementos innovadores y la capacidad de pensar fuera de lo común. Los estudiantes deben demostrar que han utilizado su imaginación para crear algo que no solo sea funcional, sino también estéticamente atractivo y diferente a lo convencional, con mensajes claros sobre la importancia de conocer los pasos de la RCP para salvar vidas.

Tecnología y mecanismos (redstone, bloques de comando...)

Este ítem se centra en el aspecto innovador del Museo. Se evaluará cómo se han utilizado tecnologías y mecanismos para mejorar la usabilidad y la eficiencia del museo. Por ejemplo, el uso de redstone para crear sistemas automatizados como puertas automáticas, iluminación y sistemas de seguridad o uso de paneles solares para que el museo sea más sostenible.

Coherencia con el tema (salas RCP)

Aquí se evalúa la **integración** de los diferentes **pasos** que hay que seguir ante una situación de **parada cardiaca** y que estarán **representadas** en las **salas** del museo que construyan los estudiantes. Es importante que se identifiquen claramente cada una de las salas con el paso que representan, además de la creatividad en la integración de elementos que representen cada una de las situaciones.

Integración de los contenidos en el museo RCP

En este ítem se evalúa la **cantidad de elementos** de contenido que han utilizado los estudiantes para que se vea claramente la importancia de la Reanimación Cardiopulmonar (RCP). Para obtener la mayor puntuación posible en este ítem de la rúbrica será necesario ver una cantidad importante de pizarras y NPCs en el museo que apoyarán con explicación de contenidos las diferentes salas de los pasos básicos de la RCP: comprobar, pedir ayuda, comprimir y desfibrilar.

Explicación de los contenidos en el mundo

Este ítem evalúa la capacidad de los estudiantes para **explicar y justificar su construcción** dentro del mundo de Minecraft. Se deberá proporcionar **descripciones claras y detalladas** de los elementos del museo, utilizando ítems como pizarras y NPCs para guiar a los visitantes. Se valorará la claridad y la organización de la información, así como la capacidad para comunicar y explicar sus creaciones.

Ejemplos de mundos en Minecraft Education

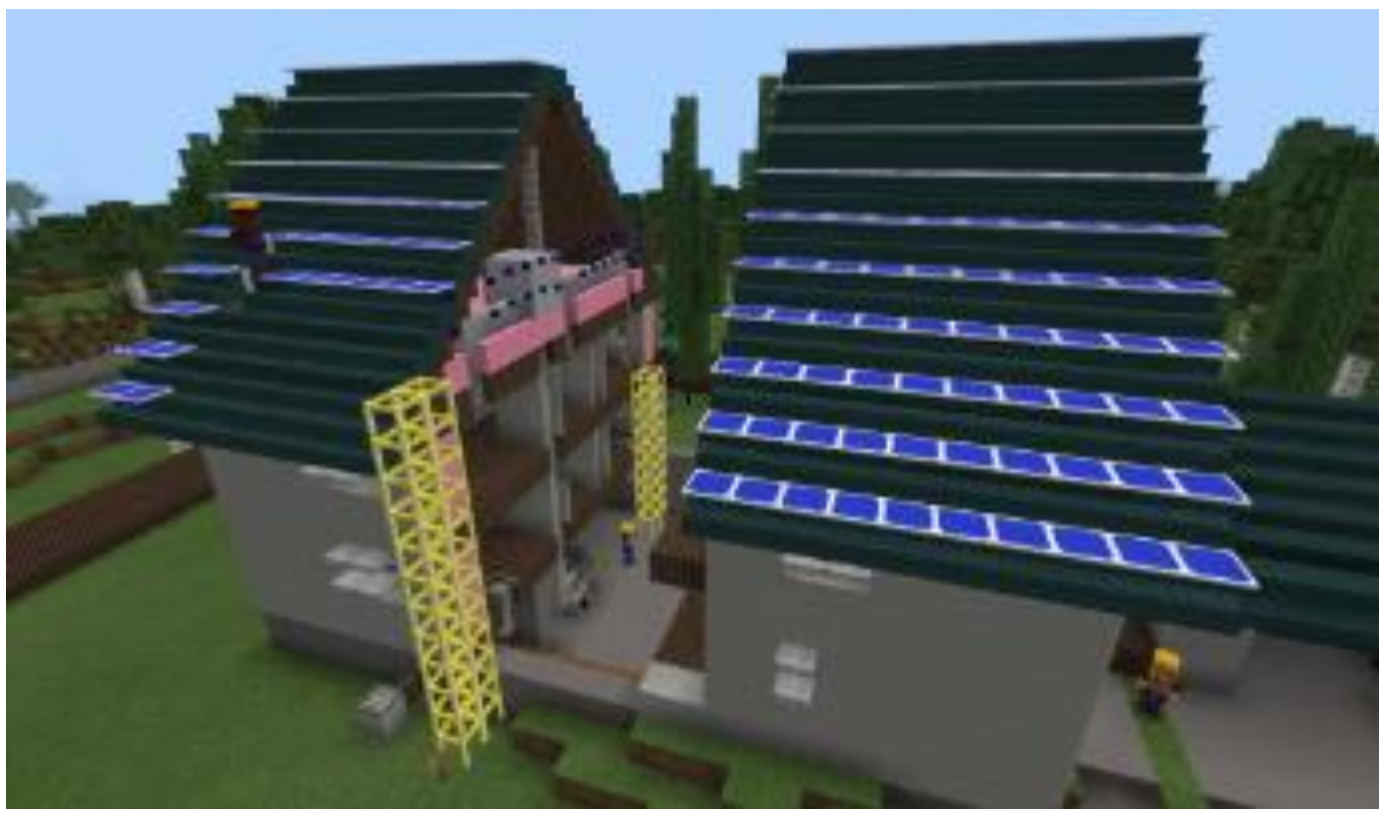

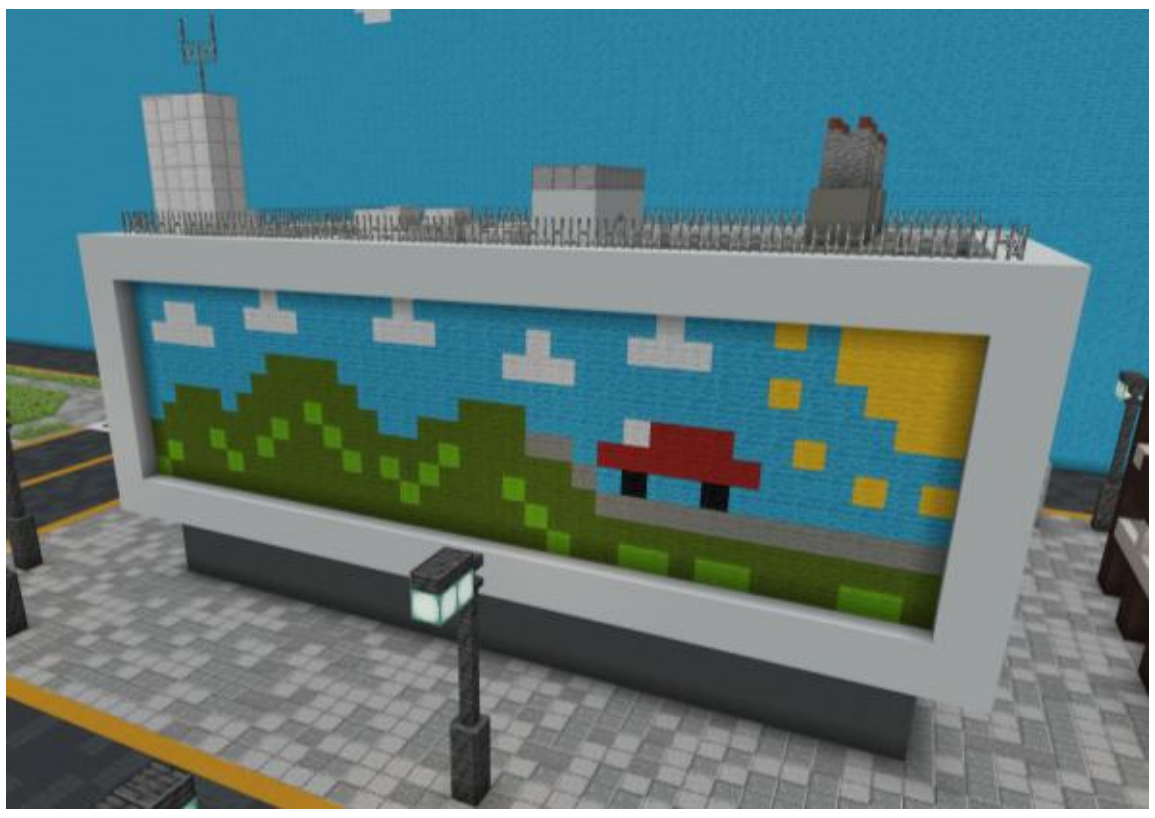

Premios

Los 3 mejores proyectos obtendrán premios exclusivos. En las redes sociales de Letcraft Educación iremos anunciando los mejores proyectos clasificados y en el evento online que tendrá lugar el 16 de octubre conoceremos los mejores proyectos.

Todos los docentes y estudiantes que participen tendrán su certificado de participación en el Challenge del Día Mundial de la Parada Cardiaca 2025.

Primer premio: 500€ en material escolar

Segundo premio: 500€ en material escolar

Tercer premio: 500€ en material escolar

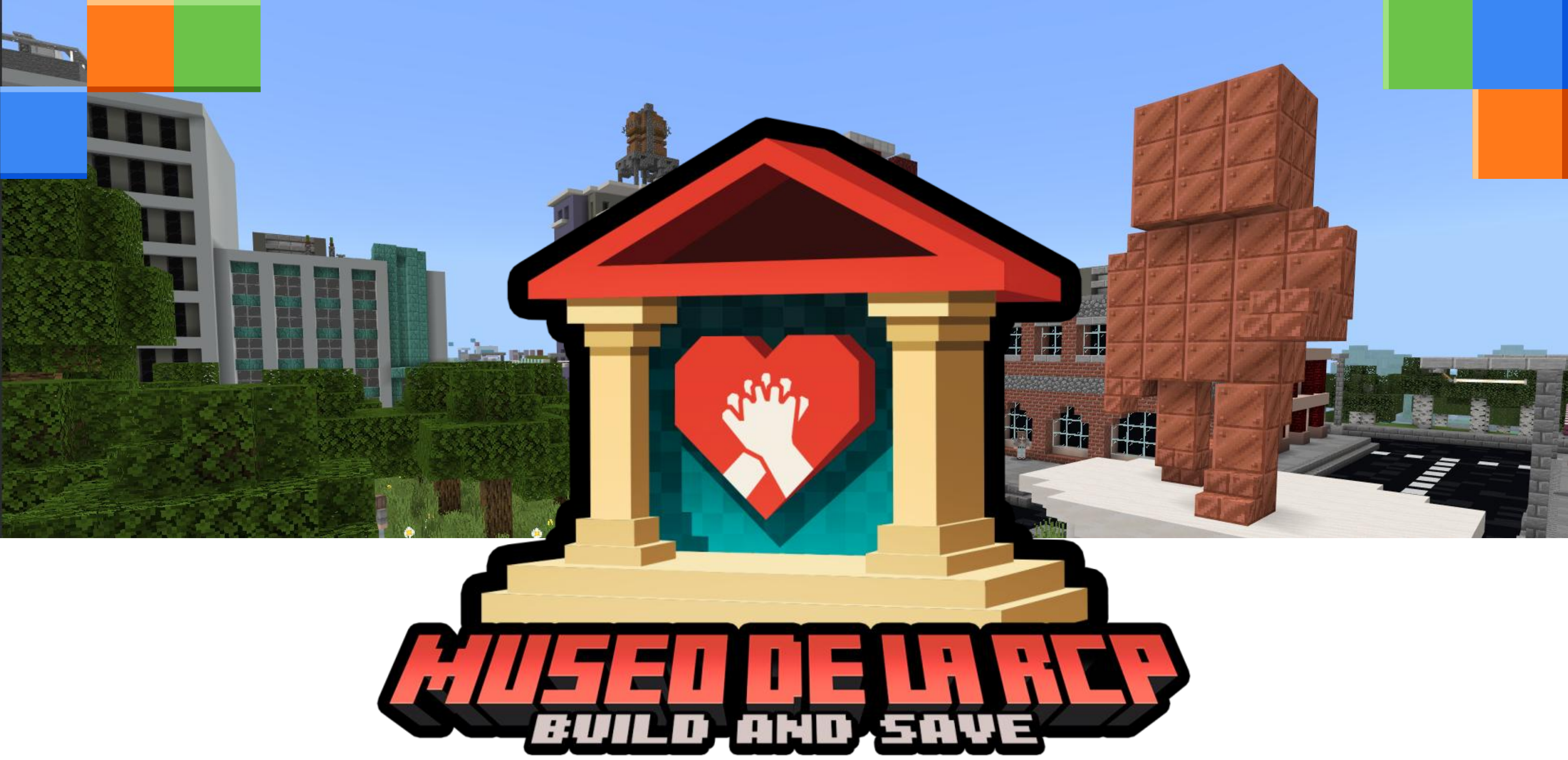

Gracias ;)

Contacto

educacion@l3tcraft.com

https://connect.l3tcrafteducacion.com/salvando-vidas-con-minecraft

Fundación MAPFRE UCAM RCP Proyecto de divulgación L3TCRAFT EDUCACIÓN
